# Gut microbial similarity in twins is driven by shared environment and aging

**DOI:** 10.1016/j.ebiom.2022.104011

**Published:** 2022-04-29

**Authors:** Ramiro Vilchez-Vargas, Jurgita Skieceviciene, Konrad Lehr, Greta Varkalaite, Cosima Thon, Mindaugas Urba, Egidijus Morkūnas, Laimutis Kucinskas, Karolina Bauraite, Denny Schanze, Martin Zenker, Peter Malfertheiner, Juozas Kupcinskas, Alexander Link

**Affiliations:** a Department of Gastroenterology, Hepatology and Infectious Diseases, Section of Molecular Gastroenterology and Microbiota-associated Diseases, Otto-von-Guericke University, Leipziger Str. 44, Magdeburg 39120, Germany; b Department of Gastroenterology, Lithuanian University of Health Sciences, Kaunas 44307, Lithuania; c Institute for Digestive Research, Lithuanian University of Health Sciences, Kaunas 44307, Lithuania; d Institute of Biology Systems and Genetic Research, Lithuanian University of Health Sciences, 44307 Kaunas, Lithuania; e Institute of Human Genetics, Otto-von-Guericke University Magdeburg, Germany

**Keywords:** Microbiome, 16S rRNA sequencing, Aging, Shared household, Stomach, *Helicobacter pylori*, Equality

## Abstract

**Background:**

Human gut microbiome composition is influenced by genetics, diet and environmental factors. We investigated the microbial composition in several gastrointestinal (GI) compartments to evaluate the impact of genetics, delivery mode, diet, household sharing and aging on microbial similarity in monozygotic and dizygotic twins.

**Methods:**

Fecal, biopsy and saliva samples were obtained from total 108 twins. DNA and/or RNA was extracted and the region V1-V2 of the 16S rRNA gene was amplified and sequenced. Bray-Curtis similarity was used for further microbiome comparisons, Mann-Whitney test was applied to evaluate the significant differences between groups and Spearman test was applied to reveal potential correlations between data.

**Findings:**

The global bacterial profiles were grouped into two clusters separating the upper and lower GI. The upper GI microbiome composition was strictly dependent on the *Helicobacter pylori* status. With a positivity rate of 55%, *H. pylori* completely colonized the stomach and separated infected twins from non-infected twins irrespective of zygosity status. Lower GI microbiome similarity between the twins was defined mainly by household-sharing and aging; whereas delivery mode and host genetics had no influence. There was a progredient decrease in the bacterial similarity with aging. Shared vs. non-shared phylotypes analysis showed that in both siblings the shared phylotypes progressively diminished with aging, while the non-shared phylotypes increased.

**Interpretation:**

Our findings strongly highlight the aging and shared household as they key determinants in gut microbial similarity and drift in twins irrespective of their zygotic state.

**Funding:**

This work was supported by the grant of the Research Council of Lithuania (Project no. APP-2/2016) and also partially supported by the funds of European Commission through the “European funds for regional development” (EFRE) as well as by the regional Ministry of Economy, Science and Digitalization as part of the “LiLife” Project as part of the “Autonomy in old Age” research group (Project ID: ZS/2018/11/95324).

**Research in context**

**Evidence before this study**

Previous studies in twins have suggested host genetics as a determinant factor for the gut microbiome profile. More recent evidence suggests an important contributing role of environmental factors on the gut microbiota structure in twins.

**Added value of this study**

We provide the evidence of the key impact of *Helicobacter pylori* on composition of stomach microbiome in twins subjects, which was independent of zygosity status. We show that similarity of gut microbiome in twins was dependent of several factors. Shared household and aging were among the most important factors in defining the gut microbiome similarity in mono- and dizygotic twins. The increasing shift of microbiome concordance with age suggests a dynamic age-and environment-related process.

**Implications of all the available evidence**

Our study support the increasing evidence that shared household, including environment and diet, as well as aging are the key dynamic factors in shaping microbiome in twins’ subjects. The data from this work may be applicable to microbiome dynamics in household members irrespective of genetic similarity.

Alt-text: Unlabelled box

## Introduction

The gut microbiome plays a crucial role in human health, and dysbiosis is related to a variety of diseases.^1^ Gut microbiome is considered an intestinal dynamic organ where its composition evolves throughout the lifetime,^2^^,^^3^ and reduction and alteration of microbial diversity is linked to gastrointestinal (GI) and non-GI diseases.^4^ Individual microbiome is variable and multiple factors contribute to bacterial composition in humans. Delivery mode,^5^^,^^6^ diet,^6^^,^^7^ physical activity^8^^,^^9^ and shared household^10^ are among the factors that have been most consistently linked with microbiome diversity.

Recently, an interplay between host genetics and microbiome has caused great interest.^11^ Several studies have analyzed the link between gut microbiome composition and host genetic variations, using a genome-wide association study (GWAS).^10^^,^^12^, ^13^, ^14^ Although a number of loci identified were associated with microbial taxonomies, microbial pathways, and measures on difference in the composition of microorganisms, most of these findings were population specific and difficult to replicate. In general, GWAS studies suggest that only up to 10% of microbial diversity might be associated with heritable factors. However, study design, population cohort and methodological approach may vary, therefore, making direct comparison challenging. Hence, additional studies are needed to determine the interplay of genetics and microbiome as it remains considered that heritable factors may determine the diversity of human microbiome.^15^

Studies in twins provide an excellent model to explore the influence of heritable factors in relation to other factors in particular to environmental factors.^16^ Several studies with focus on microbiome in twins have been completed so far.^17^, ^18^, ^19^, ^20^ Analysis of fecal specimens from monozygotic (MZ) and dizygotic (DZ) twins and their mothers revealed similarity in microbiome pattern in family members which was associated with either leanness or obesity.^17^ Another study including 416 twin pairs suggested a link between host genetics to several bacterial taxa, although the overall degree of similarity between MZ and DZ twins might be of the marginal significance.^19^ The largest cohort so far with 1,126 UK twin pairs observed an association between fecal microbiome taxa and genes related to diet, metabolism and olfaction.^18^ Nevertheless, the exact role of environmental factors is relatively unexplored in twin cohorts, and the impact of shared household and aging, although suggested, has not been proven so far. Furthermore, gastric community has been increasingly studied, but the role of genetic factors has been not explored yet. *Helicobacter pylori (H. pylori)* Infection, which is key pathogen for peptic ulcer development and gastric cancer and which has a profound influence on the microbial composition of the stomach as *H. pylori* infection kind of suppress other bacterial communities.^21^ But also, *Fusobacterium nucleatum* (*F. nucleatum*) has been frequently found in stomach of patients with gastric cancer and was associated with prognosis of gastric cancer.^22^ Microbial community has not been systematically studied taken to account the genetics in twin cohorts.

The aim of our study was to comprehensively analyze the role of genetics, delivery mode, diet, household sharing and aging on microbial similarity in saliva, and gastric mucosa, with specific attention to *Helicobacter pylori* and most specifically fecal specimens in MZ and DZ twins.

## Material and methods

### Study population

**Figure 1:**
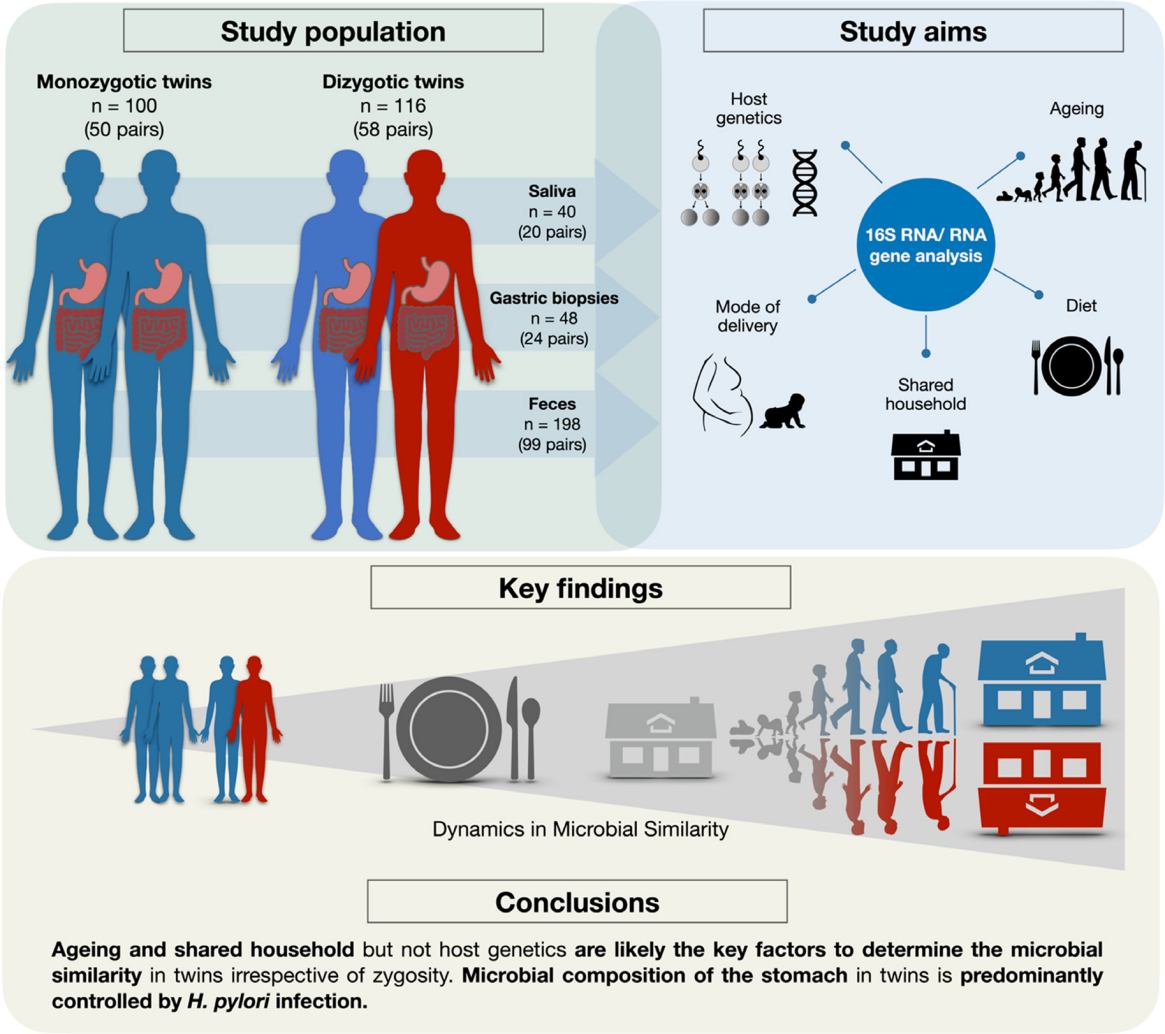
Graphical abstract. Design of the study.

Twins from the Twin Registry Center at Lithuanian University of Health Sciences were invited to take part in the study during the years 2016-2018. Detailed characteristics of the study participants are provided in Supplementary file 1. The initial study cohort included 115 twin pairs (230 subjects). 108 out of 115 twin pairs were included in the final analysis: 50 pairs of monozygotic and 58 pairs of dizygotic (29 same-sex pairs and 29 mixed-sex pairs) twins. None of the study participants has previously undergone anti-*H. pylori* treatment, and did not use PPIs or antibiotics at least one month prior to inclusion. If both twins had any potential clinical symptoms, they were offered to undergo upper gastrointestinal endoscopy. *H. pylori* presence/absence was identified after histological evaluation (haematoxylin/eosin and Giemsa staining). Demographic and diet data were collected using questionnaires (detailed description is provided in the Supplementary file 2). The study protocols were approved by Kaunas regional ethics committee (Protocol No: BE-2-10 and P1-52/2005). All participants provided a written informed consent to take part in the study. Graphical Abstract on the study design is shown in Figure 1.

### Sample collection

EDTA blood samples for DNA analysis were collected from all twins (108 paired twins, 216 samples) within this study in order to confirm their zygosity. Feces from 99 paired twins (198 samples) were collected in stool collection containers with stabilizer (Stratec molecular), delivered to laboratory within 24 h period, aliquoted and stored at -80°C until DNA extraction. Saliva samples were collected from 20 twins (40 samples) before gastroscopy procedure (08:00 am–12:00 pm). Participants were not exposed to food for at least 8 h or more and beverages for at least 4 h. Each subject rinsed their mouth with water and saliva was collected in standard 50 ml sterile conical polypropylene tube (Falcon) using passive drool collection method (collected volume 3-5 ml). After collection, the saliva samples were immediately centrifuged and pellet was stored -80C before extraction. Two biopsies from the antrum and two biopsies from the corpus were collected during upper GIi endoscopy in twins, snap frozen in liquid nitrogen and stored at -80°C until DNA (from 19 twin pairs (38 samples) for corpus and 20 from antrum) or RNA (22 twin pairs from corpus and 24 from antrum) extraction. Detailed list of biological samples collected from each twin is shown in Supplementary file 3.

### Determination of zygosity

Zygosity testing (MZ versus DZ) was performed on blood DNA samples. Short tandem repeat polymorphic DNA markers were amplified by PCR using AmpFLSTR® Identifiler® Plus PCR Amplification Kit (Thermo Fisher Scientific, USA), labeled with fluorescent markers and separated by capillary electrophoresis to distinguish different alleles at each of 15 different loci (D8S1179, D21S11, D7S820, CSF1PO, D3S1358, TH01, D13S317, D16S539, D2S1338, D19S433, vWA, TROX, D18S51, D5S818, and FGA).

### Nucleic acids extraction

Bacterial genomic DNA from stool was extracted using QIAamp Fast DNA Stool Mini Kit (Qiagen), DNA from saliva was extracted using QIAamp DNA mini kit (Qiagen) and DNA and RNA from gastric biopsy samples were extracted using Allprep DNA/RNA mini kit (Qiagen) according to the manufacturer's instructions. On-column DNA digestion during RNA purification was performed using RNase-Free DNase Set (Qiagen). RNA was reverse transcribed to cDNA using Superscript IV First-Strand Synthesis System (Thermo Fisher Scientific).

### Library construction and sequencing analysis

Library construction and sequencing of the samples were performed at the Otto-von-Guericke University Hospital of Magdeburg. Amplicon libraries were generated by amplification of the V1-V2 region of the 16S rRNA, taking as template either the DNA (16S rRNA gene) or the cDNA (16S rRNA) after 20 cycles PCR reaction, using the 27F and 338R primers, and sequenced on a MiSeq (2 × 250 bp, Illumina, Hayward, California, USA).^23^^,^^24^

FastQ files were analyzed using the dada^2^ package,^25^ version 1.10.1, in R. Overall, 11 770 078 paired-end reads were obtained, with a minimum of 39 and average of 23 446 per sample. Samples that did not reach 4 000 reads were discarded from further analysis. All samples were resampled to equal sequencing depth of 4838 reads using the phyloseq package,^26^ referring to 16 421 phylotypes (see online Supplementary file 4). Phylotypes were annotated to a taxonomic affiliation based on the naïve Bayesian classification^27^ with a pseudo-bootstrap threshold of 80%. Microbial communities were analyzed at all taxonomy ranks (from phylum to genus) and phylotype taxonomy ranks. The relative abundances (expressed as percentages) were used for downstream analyses.

Dendrograms and PCoA were built using the sample-similarity matrix by Bray-Curtis algorithm (1000 bootstrap) at phylotype level and plotted using Past,^4^ Mega^7^ software or iTol Interactive Tree of Life website.^28^ Samples were analyzed for outliers in their Bray-Curtis-Similarity, using the ROUT method (Q = 1%) in Prism 7 (GraphPad Software, La Jolla, CA). Mann-Whitney test was applied to evaluate the significant differences between groups defined a priori and correlations were performed using Spearman test using Prism 7 (GraphPad Software, La Jolla, CA). PERMANOVA was calculated using Primer-e (Primer 7, Version 7.0.17, Add on: Permanova+).^29^ *P* value < 0.05 was considered statistically different. For comparison of paired twins, we considered only the samples where both twins from the pair were successfully sequenced (198 samples out of 222 samples).

### Heritability calculations

Heritability of bacteria showing similarity between the twins on genus level was estimated as previously described.^18^ Bacteria had to be present in more than 50% of individuals. Relative abundances were transformed using Box-Cox transformation (PowerTransform command implemented in the R package ‘car’ was used to calculate λ) and regressed using multiple linear regression to eliminate influence from the number of sequencing reads per sample, age, gender, household sharing status. The residuals from this regression were then used for the heritability estimates. Heritability estimates were calculated by a twin-based ACE model using the R package ‘OpenMx’. ACE, CE, AE, and E models were calculated and p values were determined by using the likelihood ratio test in order to evaluate the significance of additive genetic (ACE vs. CE), common environment (ACE vs. AE) and unique environment (ACE vs. E) components. Multiple testing correction for 15 traits was applied for the heritability analysis using the Benjamini-Hochberg algorithm in R. Intraclass correlation coefficients (ICCs) were calculated using ‘icc’ command from the R package ‘irr’.

### Role of funding source

Funders had no role in study design, data collection, data analyses, interpretation, or writing of report.

## Results

### General cohort

**Figure 2:**
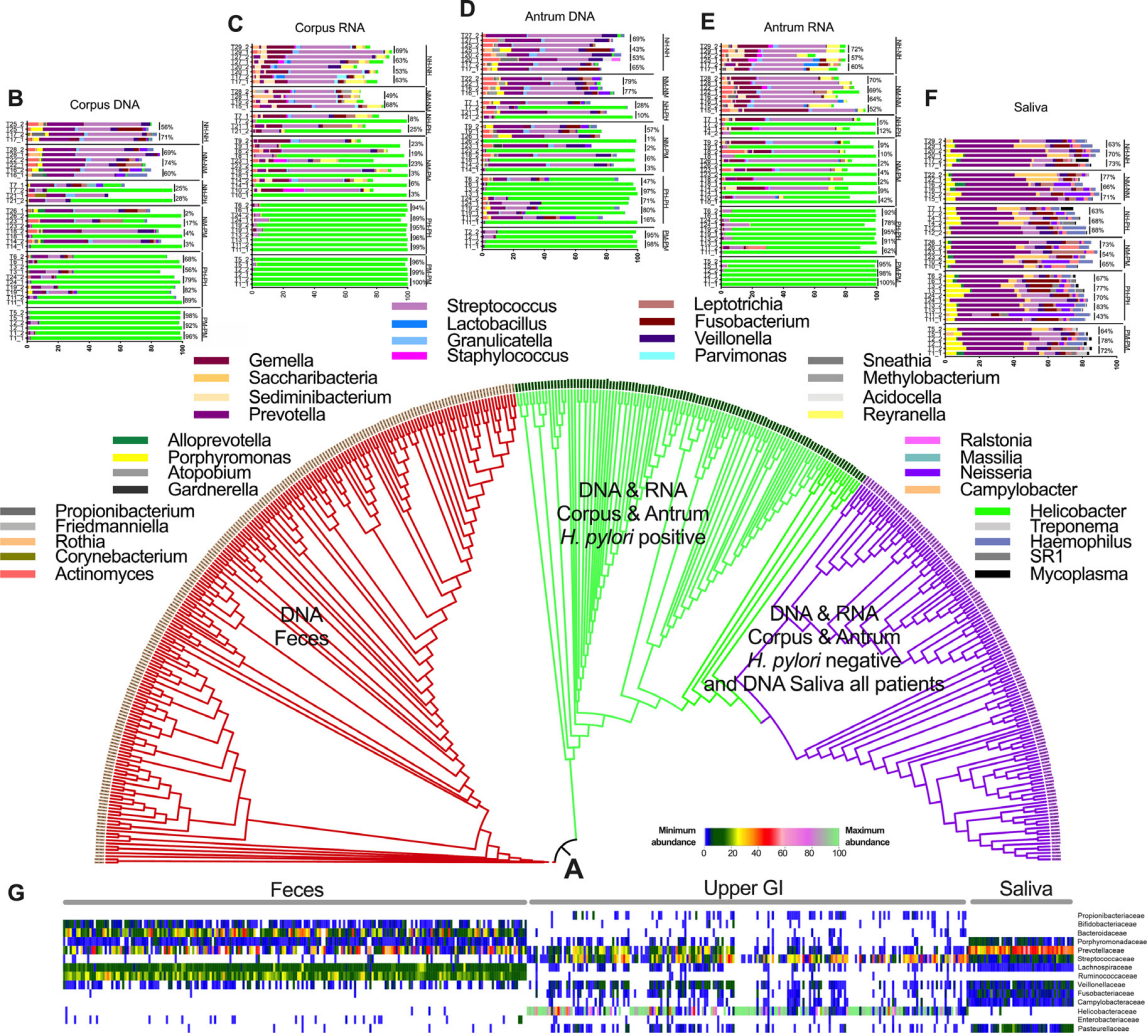
A. Group-average agglomerative hierarchical clustering of studied samples, based on the global bacterial profile at phylotype-level along the upper GI (saliva, corpus and antrum) and fecal samples. B to F: Most abundant genera detected in corpus, antrum and saliva in H. pylori negative (N) and positive (P) individuals as well as in dizygotic (DZ) and monozygotic (MZ) twins. Percentages shown the Bray-Curtis similarities between twin pairs. G: Heatmap at family-level with the most abundant taxa representing the microbiome in saliva, antrum, corpus and feces.

Bacterial communities of a total of 408 samples were characterized from 108 paired twins. The general cohort included 198 fecal samples from 99 paired twins and 210 samples from 19 to 24 twins of the upper GI, including samples from saliva (DNA), corpus (DNA and RNA) and antrum (DNA and RNA). After sequencing and rarefying library size to the minimum sequencing depth, 16 421 phylotypes, belonging to 21 different phyla and 385 genera, were retrieved and taxonomically annotated (Supplementary file 4). The global bacterial profiles were grouped into two clusters based on their % of Bray-Curtis similarities (Figure 2A), clearly showing differences between lower GI (feces) and upper GI (biopsies from corpus or antrum and saliva). The upper GI (corpus and antrum biopsy samples) microbiome composition was defined by *H. pylori* status. *H. pylori* completely colonized the stomach (Figure 2A–F) and thereby, infected twins were grouped separately from *H. pylori* negative twins. Global bacteria composition analysis along the GI indicates that saliva mainly is colonized by Prevotellaceae while stomach is colonized by Streptococcaceae and lower GI is colonized by Lachnospiraceae and Ruminococcaceae (Figure 2G).

### Helicobacter pylori status in twins

**Figure 3:**
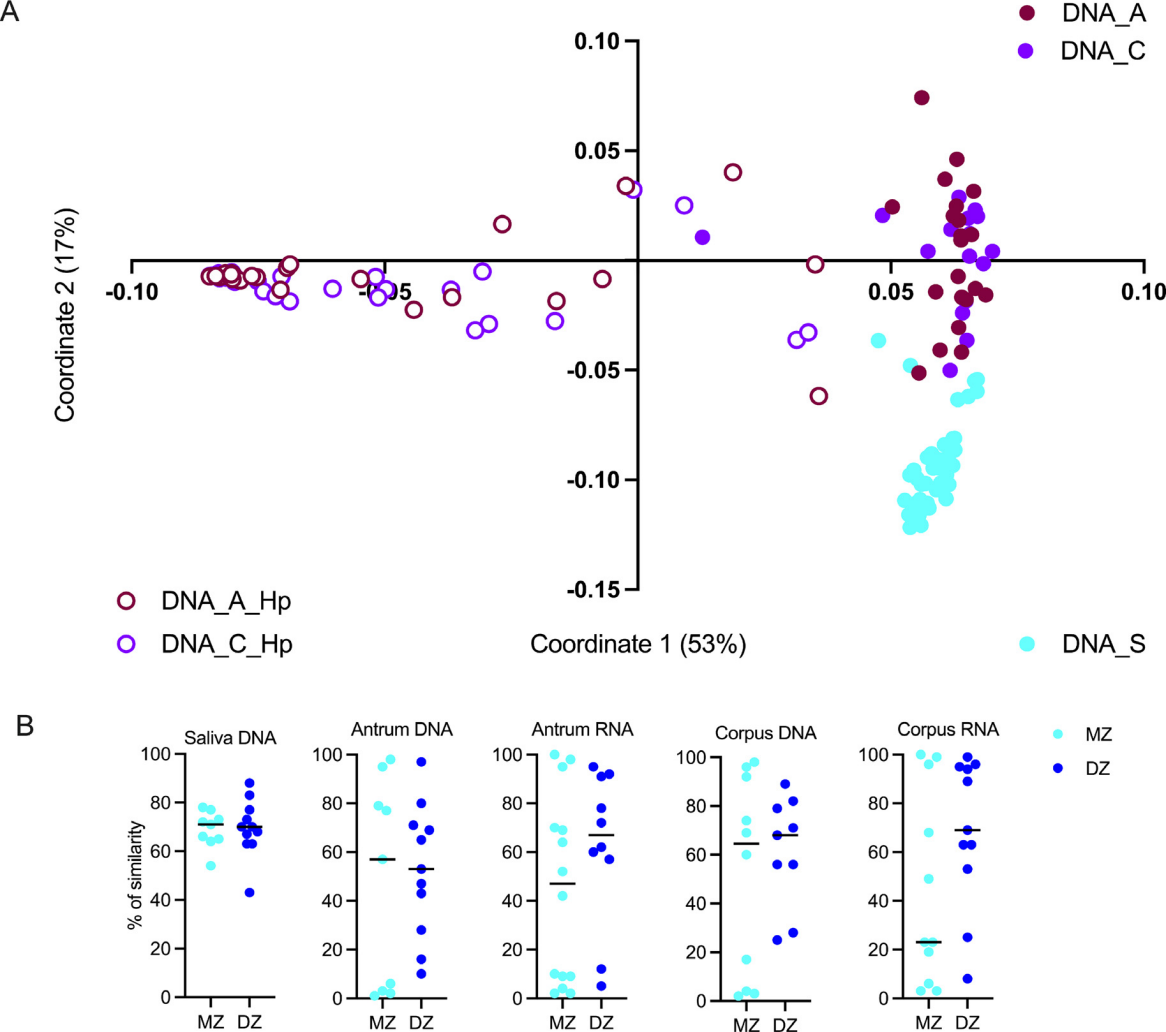
A. Principal Coordinates Analysis (PCoA) of the bacterial communities in the upper GI (DNA_S for saliva, DNA_C or RNA_C for corpus DNA_A or RNA_A for antrum) at phylotype-level based on the Bray-Curtis similarity matrix. Samples from corpus and antrum of patients infected by *H. pylori* are denoted as Hp. B: Percentage of Bray-Curtis similarities of the bacterial communities in twin pairs in saliva corpus and antrum between monozygotic twins (MZ) and dizygotic twins (DZ).

Overall, 55% of the twins with available gastric tissue were *H. pylori* positive (Figure 2B to 2E). Most of the twin pairs were concordant (both twins in the pair) for the *H. pylori* infection and eight twin pairs, incl. T4_2, T8_1 or T9_1, were discordant. As previously described, *H. pylori* was solely detected in the stomach samples.^30^ Principal coordinates analysis showed *H. pylori* to be the major determinant for differentiating twins based on their bacterial composition in the stomach (Figure 3A). Moreover, *H. pylori* infection status was similar both in MZ or DZ twins. When comparing the percentage of Bray-Curtis similarities between paired twins, no significant differences were found between MZ and DZ twins (Figure 3B, Supplementary file 5), suggesting that the genetic background does not affect the stomach bacterial community structure, although, due to the small cohort size, this statement might not be generally correct.

### Heritability of the predominant microbes in the gastrointestinal tract

Following the previous studies in twins, we calculated heritability of the most abundant taxa detected in our cohort as well as the shared environmental influence and the nonshared environmental influence applying the ACE model (Supplementary file 6).^18^^,^^19^ In order to eliminate influence from the known cofounding factors, taxa abundances were regressed on covariates (number of sequences reads per sample, age, gender, household sharing status). Results of the regression analysis are presented in the Supplementary file 7. The residuals from this regression were then used for the heritability estimates. Contrary to what was previously published, none of the fifteen more abundant genera (including *Bacteroides, Blautia, Prevotella, Bifidobacterium* among others) appeared to be significantly heritable, based on the additive genetic influence from the ACE model, although similar values were obtained for *Blautia, Faecalibacterium, Dialister* among others (Supplementary file 8). In the same manner, no influence of the shared environment on any of the genera was detected. However, regarding the nonshared environment, *Bacteroides, Blautia, Collinsella* and *Holdemanella* were strongly influenced (*p* value < 0.001) and in less extent also *Alistipes, Ruminococcus, Prevotella* and *Catenibacterium* (*p* value < 0.05). These results might suggest a stronger influence of the environment rather than the genetic background in certain taxa.

### Bacterial community assemblages in the GUT of twins

**Figure 4:**
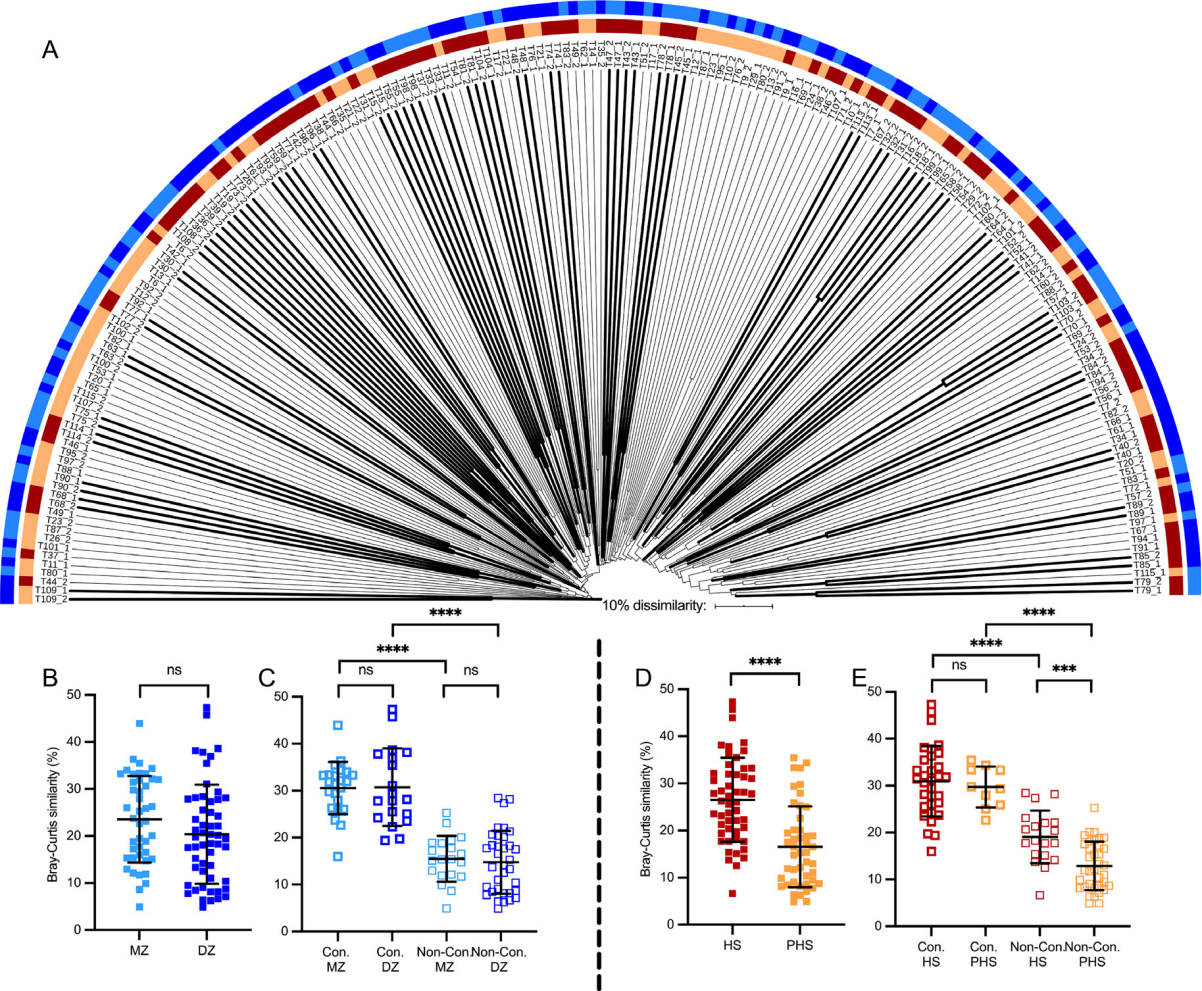
A. Group-average agglomerative hierarchical clustering of 198 fecal samples at phylotype-level. Concordant twins (Con) are denoted in bold in contrast to non-concordant (Non-Con). Light blue and dark blue denoted monozygotic (MZ) and dizygotic (DZ) twins, respectively as well as light brown and dark brown denoted shared and non-shared household twins, respectively. B to E: Bray-Curtis similarities of the bacterial communities in twin pairs and a priori defined groups (one dot represents two twins). Statistically differences are shown as *** if *p* value < 0.001 and **** if *p* value < 0.0001 and ns denotes no statistical differences.

All possible paired analyses between fecal samples (total 19 503 possible comparisons) of twins (198 samples from 99 paired-twins) revealed an average of 11±5 % of Bray-Curtis similarity, suggesting a low similarity between samples. Nonetheless, roughly 50% of the twins from a pair were grouping together and showed a higher similarity (higher than roughly 20% of similarity) to each other than with the other twin pairs. Those twins were named “concordant twins”. In contrast, twin pairs with lower similarity values (lower than roughly 20% of similarity) and not clustering together - “non-concordant twins”. Further, the possible factors affecting bacterial community structure were evaluated (Figure 4A). It was observed that 76% of concordant twins were sharing the household (HS group vs. 24% past HS, *p* value < 0.0001) (Figure 4D), while 55% of the concordant twins were MZ vs. 45% DZ (*p* > 0.05) (Figure 4B and C). This suggests that environmental factors in particular HS have much stronger influence on the similarity of the bacterial communities rather than genetic background (Figure 4A). Moreover, concordant twins living together had the highest similarity of bacterial communities (mean of similarity 31%), whereas not-concordant twins living apart – the lowest (mean of similarity 12.9%) (Figure 4E).

### Influence of nutrition on bacterial communities in the paired-twins

**Figure 5:**
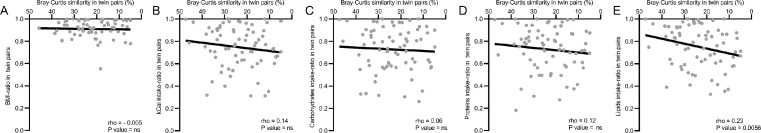
Spearman correlations (rho) between Bray-Curtis similarities of the bacterial communities in twins pairs (one dot represents two twins) and the body mass index ratio (BMI-ratio) (A), Kilocalories (Kcal) intake ratio (B), and carbohydrates, proteins (C) and lipid (D) intake ratios, respectively.

Having shown the relevance of the shared household for similarities of the bacterial communities in paired twins, we next evaluated the potential impact of diet. Out of the 99 twin pairs, records of nutritional information were available for 73 twin pairs (74% of the cohort). Based on the Spearman correlation index, diet was not the major determinant defining the bacterial community differences between the paired twins. There were no correlations between Bray-Curtis similarity and body mass index-ratio (Figure 5A), as well as kilocalories intake-ratio (Figure 5B). However, the nutrient analysis revealed that lipid intake had influence on bacterial community similarity (Figure 5E). In twins with similar intake of lipids, the bacterial communities were similar in both siblings (*p* < 0.01). Neither carbohydrate or protein intake showed statistically significant influences on the Bray-Curtis similarity although the trends were similar to the lipid's intake (Figure 5C to E).

The body mass index did not show statistically significant differences with the similarities of the bacterial communities between twins. The similar intake of total calories resulted in more similar communities within twins.

### Influence of aging on the bacterial composition between twins

**Figure 6:**
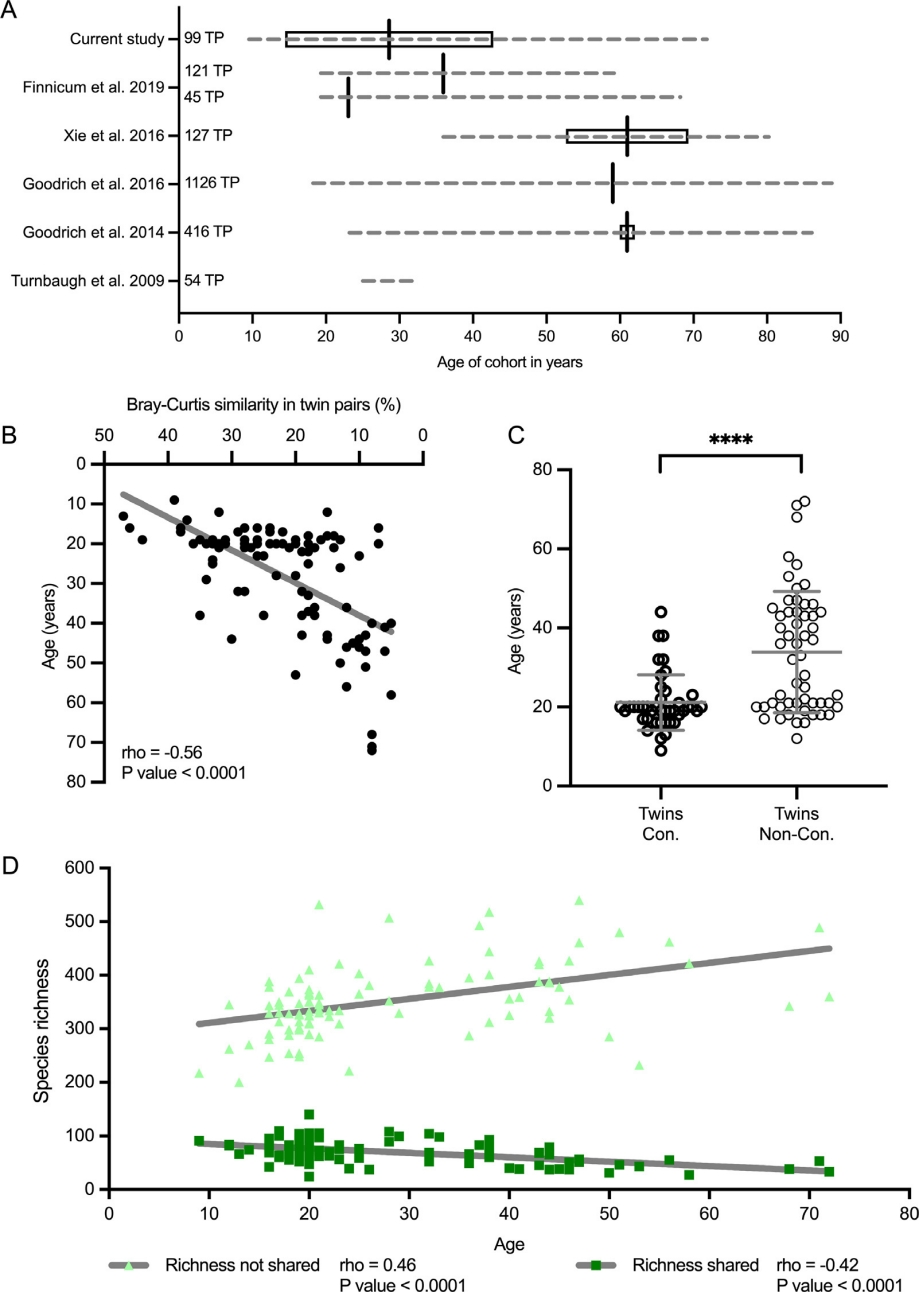
A. Overview of the age on the different cohort of twins published so far compared to this study. If it was published, for each study is shown the minimum and the maximum of age, as well as the standard deviation and the median. B: Spearman correlation (rho) in twin pairs (one dot represents two twins) between their age and their percentage of similarity of the bacterial communities. C: Differences on the age between concordant (Con) twins and non-concordant (Non-Con) twins according to the Bray-Curtis similarities shown in Fig. 4. **** denotes *p* value < 0.0001. D: Spearman correlation (rho) between the number of phylotypes shared (light green) and non-shared (dark green) between twins paired (TP).

Limited knowledge exists on the role of aging in twin cohorts as it has not been sufficiently addressed in previous twin studies. Our cohort consists of 198 twins with age ranging from 9 to 72 years (aver. age 29 ± 14 years), therefore our twin-cohort covered the broadest age-range with the highest standard deviation of all cohorts published so far (Figure 6A).

Analysis of age influence on similarities of the bacterial communities between twins revealed a strong negative correlation (Figure 6B), thereby indicating that bacterial communities diverge with increasing age in the twins (*p* < 0.0001). In addition, the analysis of concordant and non-concordant twin groups showed that concordant twins were much younger (Figure 6C), indicating the age as one of the major determinant factors in the bacterial community development. Analysis results revealed that 21 years could be considered as the age when bacterial communities diverge within paired twins. Further, shared vs. non-shared phylotype analysis showed that phylotypes shared by both siblings progressively diminished (rho = - 0.42, *p* < 0.0001) with the age, while the richness of the non-shared phylotypes strongly increased between 10 and 20-year-old twins and afterwards the increase was slower (rho = 0.46, *p* < 0.0001) (Figure 6D). In addition, when considering age as a co-variable of household sharing, age showed a p value of 0.0001 while household sharing showed a p value of 0.04 (Supplementary file 9).

### GUT taxonomy profile in twins

**Figure 7:**
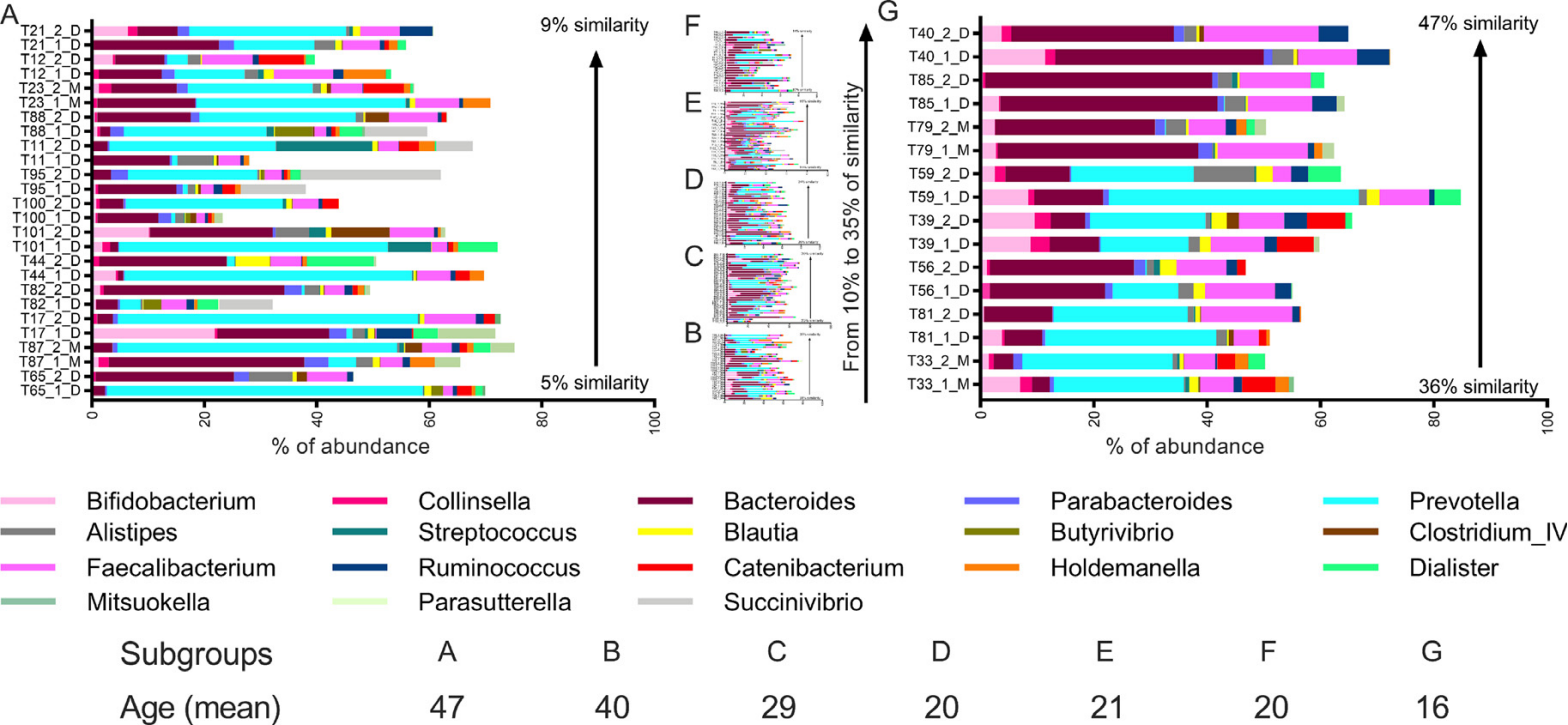
Abundances of the most predominant genera detected in fecal samples. Samples are sorted in increasing percentage of Bray-Curtis similarity and in twin pairs side-by-side, as well as divided in 7 groups due to space limitation, showing magnified the first group (A) and the last group (G) and in small sizes groups B to F.

The abundances of the most predominant genera detected in the GUT of paired twins are shown in Figure 7. Overall, eighteen genera were broadly colonizing the GUT of our cohort, *Prevotella* or *Bacteroides* being the most abundant ones. These eighteen genera made up more than 50% of the bacterial communities in most of the individuals. Colonization of the GUT by *Bacteroides* and *Prevotella* seems to be aleatory, which might suggest that either *Prevotella* or *Bacteroides*, but never both together, colonized the GUT. Interestingly, MZ twins showed a higher correlation with several genera compared to DZ twins, including *Bacteroides* (p value in MZ < 0.001), *Prevotella* (p value in MZ < 0.001), *Parasutterella* (p value in MZ < 0.01), among others (Supplementary file 8). It is worth mentioning that no correlations have been observed with any of the genera regarding delivery mode and breastfeeding (Supplementary file 10).

## Discussion

It has been hypothesized that host genetics may determine the composition of the gut microbiome,^18^ even though the genetic inheritance of microbial composition has been recently questioned.^10^ In this work, we performed systematic analysis of microbial composition of MZ and DZ twins to delineate the interplay between genetic and various concomitant factors including shared environment, delivery mode and diet in various compartments of the gut. The analysis of the stomach microbiota between MZ and DZ twins highlights the role of *H. pylori* in defining bacterial composition of the stomach mucosa. Evaluation of the fecal microbiome revealed a very strong impact of shared household and age as the main determinants of the microbial concordance among twins, which was in addition independent of the zygosity, diet and delivery mode at birth.

The role of host genetics in shaping microbiome has been suggested earlier^20^; however, more recent evidence emphasizes the predominant role of environmental factors, (such as age, sugar consumption, toothbrushing habits) over the host factors in defining the bacterial composition in twins.^31^, ^32^, ^33^ The concept of gastric microbiome is still evolving,^34^^,^^35^ with an ongoing discussion about what constitutes a gastric microbiome.^36^ While the largest studies related to twins have been dealing with easily accessible specimens like saliva and feces, only limited knowledge was available for systematic characterization of mucosal microbiome in other body niches including stomach. In the only available study at present, the authors evaluated four pairs of twins with very heterogeneous results.^37^ In current work, we evaluated microbiome in the largest cohort (24 twin pairs) that were representative for various combinations of MZ and DZ including concordant and discordant for *H. pylori* status. Even though larger cohorts are needed to evaluate the minor effects in concordant groups, we clearly show that *H. pylori* status had a dominant impact on the microbiome composition in twins irrespective of zygosity status. Our findings are in line with previous studies where *H. pylori* is the major gastric microbiome shaping species.^21^^,^^30^ In earlier high-quality studies, familial environment and *H. pylori* status were considered as the most important risk factors for peptic ulcer development which is supported by our data.^38^ In addition, *H. pylori* status in the stomach had no impact on oral microbiome as shown for the stomach.

Twin studies offer a unique opportunity to evaluate the effect of host genetics in defining microbial profile. Initial studies indicated that host genetics may influence microbiome for instance by the means of metabolic regulation.^17^^,^^19^^,^^39^ Contrary to those results, none of the most abundant genera (including *Bacteroidetes, Prevotella, Bifidobacterium*, etc.) seems to be significantly heritable in our cohort using ACE model, although some effect was visible taking into consideration the nonshared environment (*Bacteroides, Blautia, Collinsella* and *Holdemanella*). These studies indicated that although MZ twin pairs generally have more similar microbiomes compared to DZ twin pairs or unrelated individuals, MZ twins can display a large range of within-twin-pair microbiome diversity.^19^ These data suggest that environmental factors play the predominant role rather than host genetics in shaping fecal microbiome.

As of today, only a modest link between microbiome and heritability has been shown; therefore; it is likely that other factors including shared household, delivery mode or even aging might be responsible for microbial similarity in twins. A twin study from the TwinsUK registry showed that the Bray-Curtis distance between twin pairs did not associate with age.^39^ On the other hand, this study found that Bray-Curtis distance negatively correlated with the age when twins started living apart and to a lesser extent positively correlated with years the twins lived apart.^39^ Rothshild et al. have recently re-evaluated the impact of genetics and environment in a cohort of 1046 healthy Israelis and re-analyzed the link between microbiome, genetics and environment in the 2252 twins from the TwinsUK cohort.^10^ The authors show that gut microbiome composition is predominantly shaped by environmental factors while the association with individual SNPs that was previously reported could not be replicated.

In our study, we took the advantage of the wide age distribution (age range: 9–72 years) and detailed characterization of the twins and therefore were able to have an in-depth view on potential factors influencing microbiome. Neither the delivery mode, nor the breastfeeding were associated with microbial similarity in MZ and DZ twins. However, shared household was one of the most important factors that was associated with bacterial concordance, which was furthermore independent of zygosity. Our observation is also indirectly supported by a recent UK twin registry microbiome study looking at socioeconomic impacts on microbiome.^40^ The study showed that higher discordance in the index of social deprivation was associated with greater dissimilarity of twin microbiomes. Several studies have now reported that household sharing may at least partially determine microbiome similarity among relatives, while living apart reduces this similarity.^10^^,^^39^

It is important to point out that sharing a household is interlinked with other co-founding variables including diet that may additionally contribute to the microbial signature. Within our study, analysis of individual differences in the diet did not reveal any potential differences that could partially be related to common preferences of the twins. The similarity in nutritional behavior is very likely to be prone to similarity during the early life sequence. Previous analyses estimated that diet and lifestyle may be responsible for the up to 20% variance in microbiome.^10^^,^^14^ Furthermore, host lifestyle clearly affects the microbiota on the daily timescale, which is relevant for twins that share the same social and family lifestyle niche.^41^

The aging has been considered, but has only received suboptimal attention in twin cohorts’ due to difficulties to assess the lifetime frame. Our cohort consisted of MZ and DZ twins in the range of 9 to 72 years with a mean of 29.5 years, whereas, as shown on Figure 5A,the previously studied cohorts included a rather adult population with limited possibility to assess the landscape of microbial changes in twin subjects. While common in young twin pairs, Bray-Curtis similarity showed a clear shift apart in association with aging. Indeed, aging is the process that includes various steps starting from delivery mode, feeding, diseases in childhood etc. It is likely that leaving the common environment (shared household) may be a crucial step, but the drift does not stop here and shows a clear progression during the life time course.^10^^,^^42^ Our data supports recent studies showing that age, menopausal status, and prior disease were also the top factors defining the microbial urobiome diversion in twins.^43^

This work provides several important aspects on the microbiome dynamics in twins; however, various additional aspects need more accurate evaluation. Even though this work is so far the largest to assess the stomach microbiota in twins, we were unable to address the microbial similarity in depth for the substantial effect of *H. pylori* on gastric microbiota. Further studies in larger cohorts of MZ, DZ and also non-twin siblings would likely help to estimate the dynamics in microbial patterns; however, it is questionable if upper GI endoscopy in asymptomatic population may be ethically justified. This cohort included only a single time point, whereas multiple time points would be needed to assess. The dietary information was acquired using self-reported food frequency questionnaires, thereby providing possible bias in reporting certain food intake. Furthermore, this work is based on 16S rRNA; therefore, whole genome sequencing including metatranscriptomic would likely provide a much more comprehensive view on the similarity and function of the GI microbiome. A recent study in twins looked beyond the microbiome and evaluated concordance for microbiome and virome in 21 adult MZ twins showing that microbiome-discordant twins display more divergent viromes compared to microbiome-concordant twins.^44^ The results of this work were highly robust for the identified factors; therefore, we believe the data are representative also for other populations. Nevertheless, further studies with larger samples size will be necessary to delineate all compartments of microbiota, their possible functional interaction and also the way to precisely modulate microbiome in beneficial way.

## Conclusions

In conclusion, in-depth analysis of various microbial comparts of twins strongly suggest the major role of non-hereditary factors in defining the microbial similarity in twins. Among those household sharing and aging are likely the most crucial determinants of fecal microbial dynamics in twins while *H. pylori* is the key factor in defining the stomach microbiota composition.

## Declaration of interest

JS and JK were supported by the grant of the Research Council of Lithuania (Project no. APP-2/2016). PM received either speakers of consulting fees from Aboca, Bayer, Biocodex, Malesci, Mayoly-Spindler, Menarini, Synlab, Danone, Phathom during the conduct of the study. AL received research funding from EFRE (Project ID: ZS/2018/11/95324). All other authors have nothing to disclose.

## Appendix Supplementary materials

Image, application 1

Image, application 2

Image, application 3

Image, application 4

Image, Image 1

Image, application 5

Image, application 6

Image, application 3

Image, Image 2

Image, Image 3
