# Supplementary material for: Gut microbial similarity in twins is driven by shared environment and aging
Source: eBioMedicine. 2022 Apr 29;79:104011. doi: 10.1016/j.ebiom.2022.104011 (PMC9062754; doi:10.1016/j.ebiom.2022.104011)
Supplement: Supplementary file 1 [file mmc1.pdf]

Supplementary File 1. Characterization of the study cohort.

	Individuals, n	Twin pairs, n	%
Total obtained	230	115	
Total analyzed	216*	108*	
**Sex**			
male	73	NA	34
female	143	NA	66
**Age (years ± SD)**			
mean	29.5 ± 14.6	29.5 ± 14.6	
min	9	9	
max	72	72	
age ≤ 18 years	42	21	19
age > 18 years	174	87	81
**Zygosity**			
monozygotic	100	50	46
dizygotic	116	58	54
**Type of delivery**			
natural	66	33	31
CS	150	75	69

* - seven twin pairs were not included in the final analysis, because microbiome sequencing was not completed in one or both twins within a pair; NA - non-applicable; CS - Caesarian section; SD - standard deviation.
