# Supplementary material for: Gut microbial similarity in twins is driven by shared environment and aging
Source: eBioMedicine. 2022 Apr 29;79:104011. doi: 10.1016/j.ebiom.2022.104011 (PMC9062754; doi:10.1016/j.ebiom.2022.104011)
Supplement: Supplementary file 2 [file mmc2.pdf]

MITYBOS KLAUSIMYNAS

Parengtas ir išspausdintas 2012 m.
Skirtas mokslinio tyrimo dalyviams

Pagalba

Bendrosios nuorodos, kaip pildyti klausimyną

Būsime Jums dėkingi, jeigu pildydami klausimyną atkreipsite dėmesį į žemiau išvardintus dalykus:
Prašome:

- rašyti tik į įrėmintus laukelius didžiosiomis SPAUSDINTINĖMIS RAIDĖMIS,
- suklydus, tam tikrą žodį išbraukti ir rašyti į tolesnius laukelius,
- norint pateikti papildymus, įrašyti juos į paskutiniame klausimyno puslapyje specialiai tam skirtas eilutes,
- Nerašyti ant klausimyno savo vardo, pavardės.

Nuorodos, kaip pateikti atsakymus į klausimus apie tai, kokius maisto produktus Jūs vartojote per pastaruosius 12 mėnesių

Kiekvienam maisto produktui arba maisto produktų grupei yra nurodyta keletas vartojimo dažnumų.

- Iš pateiktų atsakymų prašome pasirinkti tą, kuris Jums daugiausiai tinka, ir pažymėti jį kryželiu.
- Atkreipkite dėmesį į prie kiekvieno maisto produkto nurodytus kiekius arba pateiktus piešinėlius.
- Jeigu Jūsų įprastinė porcija yra didesnė arba mažesnė už nurodytą, pažymėkite atitinkamai didesnį arba mažesnį vartojimo dažnumą.
- Atsakydami į klausimus apie Jūsų mitybą, galvokite ne tik apie maisto produktų vartojimą namuose, bet ir kitur (pvz. valgykloje, restorane arba pas draugus).
- Atkreipkite dėmesį į tai, jog klausiant apie tam tikrų daržovių ar vaisių rūšių vartojimą, turimas galvoje tik jų vartojimas sezono metu (paženklintos).

Pavyzdžiai

Pateikiame Jums klausimyno pildymo pavyzdį:
Jūs 5 kartus per savaitę valgėte grūdėtą duoną. Prašome pažymėti **„dažnai“**

Grūdėta duona, grūdėtos bandelės o niekada o retai o kartais o dažnai o visada

Kontaktai

Į Jūsų klausimus mielai atsakysime darbo dienomis nuo 8.30 iki – 16 valandos telefonu ……………………….. arba elektroniniu paštu: ....
Nuoširdžiai dėkojame už Jūsų pagalbą!

Klausimai

Bendroji dalis

Asmeniniai duomenys

Pildymo data	Diena □□<br>Mėnuo □□<br>Metai □□□□
Jūsų gimimo data	Diena □□<br>Mėnuo □□<br>Metai □□□□
Jūsų lytis	o vyras<br>o moteris
Jūsų dabartinis kūno svoris (neapsirengus)	□□□ kg
Jūsų ūgis	□□□ centimetrais

Medikamentai

Ar pastarąsias keturias savaites vartojote reguliariai medikamentus, hormonus, vitaminus arba mineralinių medžiagų preparatus, kuriuos Jums paskyrė gydytojas arba kuriuos pats nusipirkote vaistinėje (įskaitant kontraceptines tabletės, hormonų pleistrą, hormonų injekcijas, homeopatines priemones, vaistus nuo galvos skausmo ir t.t.)?	o taip<br>o ne
Nurodykite tikslų medikamento, hormono, vitaminų arba mineralinių medžiagų preparato pavadinimą bei stiprumą. Prireikus, pažiūrėkite, kas yra nurodyta ant jo pakuotės arba informaciniame lapelyje.	□□□□□□□□□□□□□□□□□□□□□<br>□□□□□□□□□□□□□□□□□□□□□<br>□□□□□□□□□□□□□□□□□□□□□<br>□□□□□□□□□□□□□□□□□□□□□<br>□□□□□□□□□□□□□□□□□□□□□<br>□□□□□□□□□□□□□□□□□□□□□

Mitybos pokyčiai

Ar šiandien Jūs maitinatės kitaip, nei prieš 12 mėnesių?	o taip<br>o iš dalies<br>o ne
Kodėl pertvarkėte savo mitybą? (pvz. Priaugote svorio, dėl viršsvorio, cukraligės, skrandžio problemų ir t.t.)	□□□□□□□□□□□□□□□□□□□□□□□□□<br>□□□□□□□□□□□□□□□□□□□□□□□□□<br>□□□□□□□□□□□□□□□□□□□□□□□□□<br>□□□□□□□□□□□□□□□□□□□□□□□□□<br>□□□□□□□□□□□□□□□□□□□□□□□□□

Vitaminai ir mineralinės medžiagos

Ar per pastaruosius 2 metus Jūs esate ne trumpiau nei mėnesį vartojęs vitaminų ir mineralinių medžiagų preparatus?	o taip<br>o ne

Prašome nurodyti, kokie tai buvo preparatai

Multipreparatai	o Multivitaminų preparatai (pvz. centrum, wellman)<br>o Mineralinių medžiagų kompleksai (pvz. Basica milteliai)	
Atskiri ir kombinuoti preparatai (prašome pažymėti visus jame esančias sudėtines dalis) (pvz. vitamino C milteliai, kalcio-magnio tabletės)	o Vitaminas C<br>o Vitaminas A<br>o Beta karotinas, karotinoidai<br>o Vitaminas E<br>o Vitaminų B kompleksas<br>o Biotinas<br>o Vitaminas B6	o Selenas<br>o Kalcis<br>o Magnis<br>o Cinkas<br>o Geležis<br>o Folio rūgštis
Kiti preparatai	o Augaliniai preparatai<br>o Baltymų koncentratai<br>o Alaus mielės, mielių dribsniai<br>o Balastinių medžiagų plytelės<br>o Sėlenos, linų sėmenys<br>o Česnakų tabletės	
Aukščiau neišvardintos medžiagos, preparatai (pavadinimas)	□□□□□□□□□□□□□□□□□□□□□□□□□<br>□□□□□□□□□□□□□□□□□□□□□□□□□<br>□□□□□□□□□□□□□□□□□□□□□□□□□<br>□□□□□□□□□□□□□□□□□□□□□□□□□<br>□□□□□□□□□□□□□□□□□□□□□□□□□	

Duona ir pieno produktai

Duona ir grūdų produktai

Duona, bandelė:<br>(1 riekė arba 1 bandelė)	o niekada<br>o 6 kartus per savaitę arba rečiau<br>o 1-2 kartus per dieną<br>o 3-4 kartus per dieną<br>o 5-6 kartus per dieną<br>o 7-8 kartus per dieną<br>o 9 kartus per dieną arba dažniau
Grūdų dribsniai (su priedais)<br>(3 valgomieji šaukštai)	o Niekada<br>o 1 kartą per mėnesį arba rečiau<br>o 2-3 kartus per mėnesį<br>o 1-2 kartus per savaitę<br>o 3-4 kartus per savaitę<br>o 5-6 kartus per savaitę<br>o 1 kartą per dieną arba dažniau

Duonos gaminiai

Balta, juoda duona, ruginės ir ruginės-kvietinės bandelės:	o niekada	o retai	o kartais	o dažnai	o visada
Grūdėta duona ir grūdėtos bandelės:	o niekada	o retai	o kartais	o dažnai	o visada
Batonas, kvietinės bandelės, sumuštinių duona (skrudinimui):	o niekada	o retai	o kartais	o dažnai	o visada
Duoniukai:	o niekada	o retai	o kartais	o dažnai	o visada

Tai, kas tepama ant duonos

Sviestas	o niekada	o retai	o kartais	o dažnai	o visada
Pusriebis margarinas	o niekada	o retai	o kartais	o dažnai	o visada
Margarinas	o niekada	o retai	o kartais	o dažnai	o visada
Lydyti taukai	o niekada	o retai	o kartais	o dažnai	o visada

Marmeladai, uogienės, vaisinės užtepėlės, medus (1 kupinas arbatinis šaukštelis)	o niekada<br>o 1 kartą per mėnesį arba rečiau<br>o 2-3 kartus per mėnesį<br>o 1-2 kartus per savaitę<br>o 3-4 kartus per savaitę<br>o 5-6 kartus per savaitę<br>o 1-2 kartus per dieną<br>o 3 kartus per dieną arba dažniau
Vegetariška užtepėlė (1 kupinas arbatinis šaukštelis)	o niekada<br>o 1 kartą per mėnesį arba rečiau<br>o 2-3 kartus per mėnesį<br>o 1-2 kartus per savaitę<br>o 3 kartus per savaitę arba dažniau

Pieno produktai (pienas ir jo gaminiai, fermentuoti pieno produktai (jogurtas, sūris ir kt.)

Kakava, vaisių skonio pieno gėrimas (150ml)	o niekada<br>o 1 kartą per mėnesį arba rečiau<br>o 2-3 kartus per mėnesį<br>o 1-2 kartus per savaitę<br>o 3-4 kartus per savaitę<br>o 5-6 kartus per savaitę<br>o 1 kartą per dieną arba dažniau
Pienas (150ml)	o niekada<br>o 1 kartą per mėnesį arba rečiau<br>o 2-3 kartus per mėnesį<br>o 1-2 kartus per savaitę<br>o 3-4 kartus per savaitę<br>o 5-6 kartus per savaitę<br>o 1-2 kartus per dieną<br>o 3 kartą per dieną arba dažniau

Natūralus jogurtas, pasukos, raugintas pienas, kefyras (150g)	○ niekada<br>○ 1 kartą per mėnesį arba rečiau<br>○ 2-3 kartus per mėnesį<br>○ 1-2 kartus per savaitę<br>○ 3-4 kartus per savaitę<br>○ 5-6 kartus per savaitę<br>○ 1 kartą per dieną arba dažniau
Vaisinis jogurtas (150g)	○ niekada<br>○ 1 kartą per mėnesį arba rečiau<br>○ 2-3 kartus per mėnesį<br>○ 1-2 kartus per savaitę<br>○ 3-4 kartus per savaitę<br>○ 5-6 kartus per savaitę<br>○ 1 kartą per dieną arba dažniau

Varškė ir sūris

Varškė, varškė su žalumynais (1 kupinas valgomasis šaukštas)	○ niekada<br>○ 1 kartą per mėnesį arba rečiau<br>○ 2-3 kartus per mėnesį<br>○ 1-2 kartus per savaitę<br>○ 3-4 kartus per savaitę<br>○ 5-6 kartus per savaitę<br>○ 1 kartą per dieną arba dažniau
Varškės sūris (riebus ir pusriebis), (1 porcija)	○ niekada<br>○ 1 kartą per mėnesį arba rečiau<br>○ 2-3 kartus per mėnesį<br>○ 1-2 kartus per savaitę<br>○ 3-4 kartus per savaitę<br>○ 5-6 kartus per savaitę<br>○ 1 kartą per dieną arba dažniau
Minkštas pelėsinis sūris (pvz. kamamberas, bri) (1 porcija)	○ niekada<br>○ 1 kartą per mėnesį arba rečiau<br>○ 2-3 kartus per mėnesį<br>○ 1-2 kartus per savaitę<br>○ 3-4 kartus per savaitę<br>○ 5-6 kartus per savaitę<br>○ 1 kartą per dieną arba dažniau
Olandiškas sūris (pvz. gouda, ementalis, edamas, Tilžės s.) (1 porcija)	○ niekada<br>○ 1 kartą per mėnesį arba rečiau<br>○ 2-3 kartus per mėnesį<br>○ 1-2 kartus per savaitę<br>○ 3-4 kartus per savaitę<br>○ 5-6 kartus per savaitę<br>○ 1 kartą per dieną arba dažniau

Vaisiai ir daržovės
Vaisiai

Obuolys, kriaušė (1 vaisius)	○ niekada<br>○ 1 kartą per mėnesį arba rečiau<br>○ 2-3 kartus per mėnesį<br>○ 1-2 kartus per savaitę<br>○ 3-4 kartus per savaitę<br>○ 5-6 kartus per savaitę<br>○ 1-2 kartus per dieną<br>○ 3 kartus per dieną arba dažniau
Apelsinas, mandarinas, kivis sezono metu (1 vaisius)	○ niekada<br>○ 1 kartą per mėnesį arba rečiau<br>○ 2-3 kartus per mėnesį<br>○ 1-2 kartus per savaitę<br>○ 3-4 kartus per savaitę<br>○ 5-6 kartus per savaitę<br>○ 1-2 kartus per dieną<br>○ 3 kartus per dieną arba dažniau
Bananas (1 vaisius)	○ niekada<br>○ 1 kartą per mėnesį arba rečiau<br>○ 2-3 kartus per mėnesį<br>○ 1-2 kartus per savaitę<br>○ 3-4 kartus per savaitę<br>○ 5-6 kartus per savaitę<br>○ 1-2 kartus per dieną<br>○ 3 kartus per dieną arba dažniau
Slyvos, persikai, abrikosai sezono metu (1 sauja)	○ niekada<br>○ 1 kartą per mėnesį arba rečiau<br>○ 2-3 kartus per mėnesį<br>○ 1-2 kartus per savaitę<br>○ 3-4 kartus per savaitę<br>○ 5-6 kartus per savaitę<br>○ 1-2 kartus per dieną<br>○ 3 kartus per dieną arba dažniau
Braškės sezono metu (1 sauja)	○ niekada<br>○ 1 kartą per mėnesį arba rečiau<br>○ 2-3 kartus per mėnesį<br>○ 1-2 kartus per savaitę<br>○ 3-4 kartus per savaitę<br>○ 5-6 kartus per savaitę<br>○ 1-2 kartus per dieną<br>○ 3 kartus per dieną arba dažniau

Raudoni serbentai, gervuogės, šilauogės sezono metu (1 sauja)	○ niekada<br>○ 1 kartą per mėnesį arba rečiau<br>○ 2-3 kartus per mėnesį<br>○ 1-2 kartus per savaitę<br>○ 3-4 kartus per savaitę<br>○ 5-6 kartus per savaitę<br>○ 1-2 kartus per dieną<br>○ 3 kartus per dieną arba dažniau
Vynuogės sezono metu (1 sauja)	○ niekada<br>○ 1 kartą per mėnesį arba rečiau<br>○ 2-3 kartus per mėnesį<br>○ 1-2 kartus per savaitę<br>○ 3-4 kartus per savaitę<br>○ 5-6 kartus per savaitę<br>○ 1-2 kartus per dieną<br>○ 3 kartus per dieną arba dažniau
Virti, konservuoti vaisiai (1 desertinis dubenėlis)	○ niekada<br>○ 1 kartą per mėnesį arba rečiau<br>○ 2-3 kartus per mėnesį<br>○ 1-2 kartus per savaitę<br>○ 3 kartus per savaitę arba dažniau
Šviežių vaisių salotos (1 desertinis dubenėlis)	○ niekada<br>○ 1 kartą per mėnesį arba rečiau<br>○ 2-3 kartus per mėnesį<br>○ 1-2 kartus per savaitę<br>○ 3 kartus per savaitę arba dažniau

Džiovinti vaisiai ir riešutai

Džiovinti vaisiai (pvz. figos, razinos, datulės) (1 sauja)	○ niekada<br>○ 1 kartą per mėnesį arba rečiau<br>○ 2-3 kartus per mėnesį<br>○ 1-2 kartus per savaitę<br>○ 3-4 kartus per savaitę<br>○ 5 kartus per savaitę arba dažniau
Riešutai (švieži, sūdyti) (1 sauja)	○ niekada<br>○ 1 kartą per mėnesį arba rečiau<br>○ 2-3 kartus per mėnesį<br>○ 1-2 kartus per savaitę<br>○ 3-4 kartus per savaitę<br>○ 5-6 kartus per savaitę<br>○ 1 kartą per dieną arba dažniau

Linų sėmenys (1 nubrauktas valgomasis šaukštas)	○ niekada<br>○ 1 kartą per mėnesį arba rečiau<br>○ 2-3 kartus per mėnesį<br>○ 1-2 kartus per savaitę<br>○ 3-4 kartus per savaitę<br>○ 5-6 kartus per savaitę<br>○ 1 kartą per dieną arba dažniau

Žalios (nevirtos) daržovės

Morkos, žalios arba virtos (2 morkos arba 1 porcija)	○ niekada<br>○ 1 kartą per mėnesį arba rečiau<br>○ 2-3 kartus per mėnesį<br>○ 1-2 kartus per savaitę<br>○ 3-4 kartus per savaitę<br>○ 5 kartus per savaitę arba dažniau
Pomidorai, žali (1 pomidoras)	○ niekada<br>○ 1 kartą per mėnesį arba rečiau<br>○ 2-3 kartus per mėnesį<br>○ 1-2 kartus per savaitę<br>○ 3-4 kartus per savaitę<br>○ 5-6 kartus per savaitę<br>○ 1 kartą per dieną arba dažniau
Žaliosios salotos, salotų mišinys, pekino salotos, baltagūžiai kopūstai, nevirti (1 porcija)	○ niekada<br>○ 1 kartą per mėnesį arba rečiau<br>○ 2-3 kartus per mėnesį<br>○ 1-2 kartus per savaitę<br>○ 3-4 kartus per savaitę<br>○ 5-6 kartus per savaitę<br>○ 1 kartą per dieną arba dažniau
Ilgavaisis agurkas (¼ vieneto)	○ niekada<br>○ 1 kartą per mėnesį arba rečiau<br>○ 2-3 kartus per mėnesį<br>○ 1-2 kartus per savaitę<br>○ 3-4 kartus per savaitę<br>○ 5-6 kartus per savaitę<br>○ 1 kartą per dieną arba dažniau
Paprika, nevirta (1 paprika)	○ niekada<br>○ 1 kartą per mėnesį arba rečiau<br>○ 2-3 kartus per mėnesį<br>○ 1-2 kartus per savaitę<br>○ 3-4 kartus per savaitę<br>○ 5 kartus per savaitę arba dažniau

Ar reguliariai valgote česnakus?	nevirtus<br>virtus, keptus, konservuotus	○ ne<br>○ ne	○ taip<br>○ taip

Virtos daržovės

Rauginti kopūstai (1 porcija)	○ niekada<br>○ 1 kartą per mėnesį arba rečiau<br>○ 2-3 kartus per mėnesį<br>○ 1-2 kartus per savaitę<br>○ 3 kartus per savaitę arba dažniau
Špinatai (1 porcija)	○ niekada<br>○ 1 kartą per mėnesį arba rečiau<br>○ 2-3 kartus per mėnesį<br>○ 1-2 kartus per savaitę<br>○ 3 kartus per savaitę arba dažniau
Baltagūžiai kopūstai, žiediniai kopūstai, brokoliai, kaliaropės (1 porcija)	○ niekada<br>○ 1 kartą per mėnesį arba rečiau<br>○ 2-3 kartus per mėnesį<br>○ 1-2 kartus per savaitę<br>○ 3 kartus per savaitę arba dažniau
Smidrai (šparagai) sezono metu (4 stiebeliai)	○ niekada<br>○ 1 kartą per mėnesį arba rečiau<br>○ 2-3 kartus per mėnesį<br>○ 1-2 kartus per savaitę<br>○ 3-4 kartus per savaitę<br>○ 5-6 kartus per savaitę<br>○ 1 kartą per dieną arba dažniau
Cukinijos (1 porcija)	○ niekada<br>○ 1 kartą per mėnesį arba rečiau<br>○ 2-3 kartus per mėnesį<br>○ 1-2 kartus per savaitę<br>○ 3 kartus per savaitę arba dažniau
Šparaginės pupelės, žali žirneliai (1 porcija)	○ niekada<br>○ 1 kartą per mėnesį arba rečiau<br>○ 2-3 kartus per mėnesį<br>○ 1-2 kartus per savaitę<br>○ 3 kartus per savaitę arba dažniau
Mišrios daržovės, daržovių mišrainė (1 porcija)	○ niekada<br>○ 1 kartą per mėnesį arba rečiau<br>○ 2-3 kartus per mėnesį<br>○ 1-2 kartus per savaitę<br>○ 3 kartus per savaitę arba dažniau

Daržovių patiekalai

Tiršta daržovių, bulvių sriuba (1 sriubos lėkštė)	○ niekada<br>○ 1 kartą per mėnesį arba rečiau<br>○ 2-3 kartus per mėnesį<br>○ 1-2 kartus per savaitę<br>○ 3 kartus per savaitę arba dažniau
Tiršta lęšių, žirnelių, pupelių sriuba (1 sriubos lėkštė)	○ niekada<br>○ 1 kartą per mėnesį arba rečiau<br>○ 2-3 kartus per mėnesį<br>○ 1-2 kartus per savaitę<br>○ 3 kartus per savaitę arba dažniau
Pomidorų padažas (taip pat konservuoti pomidorai, trinti pomidorai) (1 samtis)	○ niekada<br>○ 1 kartą per mėnesį arba rečiau<br>○ 2-3 kartus per mėnesį<br>○ 1-2 kartus per savaitę<br>○ 3 kartus per savaitę arba dažniau
Kečupas (1 nubrauktas valgomasis šaukštas)	○ niekada<br>○ 1 kartą per mėnesį arba rečiau<br>○ 2-3 kartus per mėnesį<br>○ 1-2 kartus per savaitę<br>○ 3 kartus per savaitę arba dažniau

Bulvės ir grūdų produktai

Bulvės, bulvių košė, virtų ar tarkuotų bulvių kukuliai (1 porcija)	○ niekada<br>○ 1 kartą per mėnesį arba rečiau<br>○ 2-3 kartus per mėnesį<br>○ 1-2 kartus per savaitę<br>○ 3-4 kartus per savaitę<br>○ 5-6 kartus per savaitę<br>○ 1 kartą per dieną arba dažniau
Keptos bulvės (1 porcija)	○ niekada<br>○ 1 kartą per mėnesį arba rečiau<br>○ 2-3 kartus per mėnesį<br>○ 1-2 kartus per savaitę<br>○ 3 kartus per savaitę arba dažniau
Ryžiai, makaronai (1 porcija)	○ niekada<br>○ 1 kartą per mėnesį arba rečiau<br>○ 2-3 kartus per mėnesį<br>○ 1-2 kartus per savaitę<br>○ 3-4 kartus per savaitę<br>○ 5 kartus per savaitę arba dažniau

Kiaušinis, virtas arba keptas (1 vnt.)	o niekada<br>o 1 kartą per mėnesį arba rečiau<br>o 2-3 kartus per mėnesį<br>o 1-2 kartus per savaitę<br>o 3-4 kartus per savaitę<br>o 5 kartus per savaitę arba dažniau

Mėsa ir žuvis

Mėsos gaminiai

Kepenų paštetas<br>(1 porcija)	o niekada<br>o 1 kartą per mėnesį arba rečiau<br>o 2-3 kartus per mėnesį<br>o 1-2 kartus per savaitę<br>o 3-4 kartus per savaitę<br>o 5 kartus per savaitę arba dažniau
Rūkyta dešra (pvz. saliamis)<br>(1 porcija)	o niekada<br>o 1 kartą per mėnesį arba rečiau<br>o 2-3 kartus per mėnesį<br>o 1-2 kartus per savaitę<br>o 3-4 kartus per savaitę<br>o 5-6 kartus per savaitę<br>o 1 kartą per dieną arba dažniau
Virta dešra, virta kumpinė dešra<br>(1 porcija)	o niekada<br>o 1 kartą per mėnesį arba rečiau<br>o 2-3 kartus per mėnesį<br>o 1-2 kartus per savaitę<br>o 3-4 kartus per savaitę<br>o 5-6 kartus per savaitę<br>o 1 kartą per dieną arba dažniau
Kraujinė dešra<br>(1 porcija)	o niekada<br>o 1 kartą per mėnesį arba rečiau<br>o 2-3 kartus per mėnesį<br>o 1-2 kartus per savaitę<br>o 3-4 kartus per savaitę<br>o 5-6 kartus per savaitę<br>o 1 kartą per dieną arba dažniau
Keptas ar rūkytas kumpis, rūkyta nugarinė<br>(1 riekė)	o niekada<br>o 1 kartą per mėnesį arba rečiau<br>o 2-3 kartus per mėnesį<br>o 1-2 kartus per savaitę<br>o 3-4 kartus per savaitę<br>o 5-6 kartus per savaitę<br>o 1 kartą per dieną arba dažniau

Grilio dešrelė<br>(1 vienetas)	o niekada<br>o 1 kartą per mėnesį arba rečiau<br>o 2-3 kartus per mėnesį<br>o 1-2 kartus per savaitę<br>o 3 kartus per savaitę arba dažniau
Kotletas, mėsainis, netikras zuikis (vienetas arba riekė)	o niekada<br>o 1 kartą per mėnesį arba rečiau<br>o 2-3 kartus per mėnesį<br>o 1-2 kartus per savaitę<br>o 3 kartus per savaitę arba dažniau
Virta dešrelė<br>(1 vienetas)	o niekada<br>o 1 kartą per mėnesį arba rečiau<br>o 2-3 kartus per mėnesį<br>o 1-2 kartus per savaitę<br>o 3 kartus per savaitę arba dažniau

Mėsa ir žuvis

Kepenys<br>(1 porcija)	o niekada<br>o 1 kartą per mėnesį arba rečiau<br>o 2-3 kartus per mėnesį<br>o 1 kartą per savaitę arba dažniau
Kiauliena<br>(1 porcija)	o niekada<br>o 1 kartą per mėnesį arba rečiau<br>o 2-3 kartus per mėnesį<br>o 1-2 kartus per savaitę<br>o 3 kartus per savaitę arba dažniau
Jautiena<br>(1 porcija)	o niekada<br>o 1 kartą per mėnesį arba rečiau<br>o 2-3 kartus per mėnesį<br>o 1-2 kartus per savaitę<br>o 3 kartus per savaitę arba dažniau
Paukštiena<br>(1 porcija)	o niekada<br>o 1 kartą per mėnesį arba rečiau<br>o 2-3 kartus per mėnesį<br>o 1-2 kartus per savaitę<br>o 3 kartus per savaitę arba dažniau
Silkė, tunas, skumbrė, lašiša<br>(1 porcija)	o niekada<br>o 1 kartą per mėnesį arba rečiau<br>o 2-3 kartus per mėnesį<br>o 1-2 kartus per savaitę<br>o 3 kartus per savaitę arba dažniau

Kita žuvis (pvz. upėtakis, žuvies file, žuvies lazdelės)<br>(1 porcija)	○ niekada<br>○ 1 kartą per mėnesį arba rečiau<br>○ 2-3 kartus per mėnesį<br>○ 1-2 kartus per savaitę<br>○ 3 kartus per savaitę arba dažniau

Iš kokio gyvulio mėsos pagamintas Jūsų valgytas produktas?
(Galima pateikti kelis atsakymo variantus).

Kotletas, mėsainis, netikras zuikis	○ kiauliena	○ jautiena	○ paukštiena	○ nežinau/įvairi
Grilio dešrelė	○ kiauliena	○ jautiena	○ paukštiena	○ nežinau/įvairi
Virta dešra	○ kiauliena	○ jautiena	○ paukštiena	○ nežinau/įvairi
Kepenys	○ kiauliena	○ jautiena	○ paukštiena	○ nežinau/įvairi

Apkepai ir picos

Maistingas apkepas<br>(1 porcija)	○ niekada<br>○ 1 kartą per mėnesį arba rečiau<br>○ 2-3 kartus per mėnesį<br>○ 1-2 kartus per savaitę<br>○ 3 kartus per savaitę arba dažniau
Skaidrus sultinys (su daržovėmis, su makaronais, su mėsa)<br>(1 sriubos puodelis)	○ niekada<br>○ 1 kartą per mėnesį arba rečiau<br>○ 2-3 kartus per mėnesį<br>○ 1-2 kartus per savaitę<br>○ 3 kartus per savaitę arba dažniau
Tofu (sojos proteinas, mėsos pakaitalas)<br>(1 porcija)	○ niekada<br>○ 1 kartą per mėnesį arba rečiau<br>○ 2-3 kartus per mėnesį<br>○ 1-2 kartus per savaitę<br>○ 3 kartus per savaitę arba dažniau
Užkeptas prancūziškas batonas, pica<br>(1 pica arba 2 batono puselės)	○ niekada<br>○ 1 kartą per mėnesį arba rečiau<br>○ 2-3 kartus per mėnesį<br>○ 1-2 kartus per savaitę<br>○ 3 kartus per savaitę arba dažniau
Paruoštos salotos/mišrainės (salotos su mėsa, salotos su kiaušiniais)<br>(1 kupinas valgomasis šaukštas)	○ niekada<br>○ 1 kartą per mėnesį arba rečiau<br>○ 2-3 kartus per mėnesį<br>○ 1-2 kartus per savaitę<br>○ 3 kartus per savaitę arba dažniau

Lietiniai blynai<br>(2 vienetai)	o niekada<br>o 1 kartą per mėnesį arba rečiau<br>o 2-3 kartus per mėnesį<br>o 1-2 kartus per savaitę<br>o 3 kartus per savaitę arba dažniau
Ledai, pudingas, vaisinės varškės desertas<br>(1 desertinis dubenėlis)	o niekada<br>o 1 kartą per mėnesį arba rečiau<br>o 2-3 kartus per mėnesį<br>o 1-2 kartus per savaitę<br>o 3-4 kartus per savaitę<br>o 5 kartus per savaitę arba dažniau

Pyragai ir saldumynai

Vaisinis pyragas<br>(1 gabalėlis)	o niekada<br>o 1 kartą per mėnesį arba rečiau<br>o 2-3 kartus per mėnesį<br>o 1-2 kartus per savaitę<br>o 3-4 kartus per savaitę<br>o 5 kartus per savaitę arba dažniau
Mielinė bandelė, mielinis pyragas<br>(1 bandelė arba 1 gabalėlis)	o niekada<br>o 1 kartą per mėnesį arba rečiau<br>o 2-3 kartus per mėnesį<br>o 1-2 kartus per savaitę<br>o 3-4 kartus per savaitę<br>o 5 kartus per savaitę arba dažniau
Tortas su grietinėle, kremu, vaisiais, ar sūrio pyragas<br>(1 gabalėlis)	o niekada<br>o 1 kartą per mėnesį arba rečiau<br>o 2-3 kartus per mėnesį<br>o 1-2 kartus per savaitę<br>o 3-4 kartus per savaitę<br>o 5 kartus per savaitę arba dažniau
Diabetikams skirtas pyragas, sausainiai (1 gabalėlis arba 3 sausainiai)	o niekada<br>o 1 kartą per mėnesį arba rečiau<br>o 2-3 kartus per mėnesį<br>o 1-2 kartus per savaitę<br>o 3-4 kartus per savaitę<br>o 5 kartus per savaitę arba dažniau
Plokštainis, keksas<br>(1 gabalėlis)	o niekada<br>o 1 kartą per mėnesį arba rečiau<br>o 2-3 kartus per mėnesį<br>o 1-2 kartus per savaitę<br>o 3-4 kartus per savaitę<br>o 5 kartus per savaitę arba dažniau

Plakta grietinėlė<br>(1 kupinas valgomasis šaukštas)	○ niekada<br>○ 1 kartą per mėnesį arba rečiau<br>○ 2-3 kartus per mėnesį<br>○ 1-2 kartus per savaitę<br>○ 3-4 kartus per savaitę<br>○ 5 kartus per savaitę arba dažniau
Sausainiai<br>(3 vienetai)	○ niekada<br>○ 1 kartą per mėnesį arba rečiau<br>○ 2-3 kartus per mėnesį<br>○ 1-2 kartus per savaitę<br>○ 3-4 kartus per savaitę<br>○ 5 kartus per savaitę arba dažniau
Šokoladas, šokoladiniai saldainiai<br>(50g)	○ niekada<br>○ 1 kartą per mėnesį arba rečiau<br>○ 2-3 kartus per mėnesį<br>○ 1-2 kartus per savaitę<br>○ 3-4 kartus per savaitę<br>○ 5-6 kartus per savaitę<br>○ 1 kartą per dieną arba dažniau

Gėrimai

Mineralinis vanduo, vandentiekio vanduo (200ml)	○ niekada<br>○ 1 kartą per mėnesį arba rečiau<br>○ 2-3 kartus per mėnesį<br>○ 1-3 kartus per savaitę<br>○ 4-6 kartus per savaitę<br>○ 1-2 kartus per dieną	○ 3-4 kartus per dieną<br>○ 5-6 kartus per dieną<br>○ 7-8 kartus per dieną<br>○ 9-10 kartų per dieną<br>○ 11 kartų per dieną arba dažniau
Gazuotas saldintas gėrimas (pvz. Coca cola) (200ml)	○ niekada<br>○ 1 kartą per mėnesį arba rečiau<br>○ 2-3 kartus per mėnesį<br>○ 1-3 kartus per savaitę<br>○ 4-6 kartus per savaitę<br>○ 1-2 kartus per dieną	○ 3-4 kartus per dieną<br>○ 5-6 kartus per dieną<br>○ 7-8 kartus per dieną<br>○ 9-10 kartų per dieną<br>○ 11 kartų per dieną arba dažniau
Apelsinų sultys (200ml)	○ niekada<br>○ 1 kartą per mėnesį arba rečiau<br>○ 2-3 kartus per mėnesį<br>○ 1-3 kartus per savaitę<br>○ 4-6 kartus per savaitę<br>○ 1-2 kartus per dieną	○ 3-4 kartus per dieną<br>○ 5-6 kartus per dieną<br>○ 7-8 kartus per dieną<br>○ 9-10 kartų per dieną<br>○ 11 kartų per dieną arba dažniau
Obuolių sultys (200ml)	○ niekada<br>○ 1 kartą per mėnesį arba rečiau<br>○ 2-3 kartus per mėnesį<br>○ 1-3 kartus per savaitę<br>○ 4-6 kartus per savaitę<br>○ 1-2 kartus per dieną	○ 3-4 kartus per dieną<br>○ 5-6 kartus per dieną<br>○ 7-8 kartus per dieną<br>○ 9-10 kartų per dieną<br>○ 11 kartų per dieną arba dažniau

Juodųjų serbentų sultys (200ml)	○ niekada<br>○ 1 kartą per mėnesį arba rečiau<br>○ 2-3 kartus per mėnesį<br>○ 1-3 kartus per savaitę<br>○ 4-6 kartus per savaitę<br>○ 1-2 kartus per dieną	○ 3-4 kartus per dieną<br>○ 5-6 kartus per dieną<br>○ 7-8 kartus per dieną<br>○ 9-10 kartų per dieną<br>○ 11 kartų per dieną arba dažniau
Vynuogių, vyšnių, ananasų sultys (200ml)	○ niekada<br>○ 1 kartą per mėnesį arba rečiau<br>○ 2-3 kartus per mėnesį<br>○ 1-3 kartus per savaitę<br>○ 4-6 kartus per savaitę<br>○ 1-2 kartus per dieną	○ 3-4 kartus per dieną<br>○ 5-6 kartus per dieną<br>○ 7-8 kartus per dieną<br>○ 9-10 kartų per dieną<br>○ 11 kartų per dieną arba dažniau
Multivitaminų sultys (200ml)	○ niekada<br>○ 1 kartą per mėnesį arba rečiau<br>○ 2-3 kartus per mėnesį<br>○ 1-3 kartus per savaitę<br>○ 4-6 kartus per savaitę<br>○ 1-2 kartus per dieną	○ 3-4 kartus per dieną<br>○ 5-6 kartus per dieną<br>○ 7-8 kartus per dieną<br>○ 9-10 kartų per dieną<br>○ 11 kartų per dieną arba dažniau
Morkų sultys (200ml)	○ niekada<br>○ 1 kartą per mėnesį arba rečiau<br>○ 2-3 kartus per mėnesį<br>○ 1-3 kartus per savaitę<br>○ 4-6 kartus per savaitę<br>○ 1-2 kartus per dieną	○ 3-4 kartus per dieną<br>○ 5-6 kartus per dieną<br>○ 7-8 kartus per dieną<br>○ 9-10 kartų per dieną<br>○ 11 kartų per dieną arba dažniau
Kava su kofeinu (150ml)	○ niekada<br>○ 1 kartą per mėnesį arba rečiau<br>○ 2-3 kartus per mėnesį<br>○ 1-3 kartus per savaitę<br>○ 4-6 kartus per savaitę<br>○ 1-2 kartus per dieną	○ 3-4 kartus per dieną<br>○ 5-6 kartus per dieną<br>○ 7-8 kartus per dieną<br>○ 9-10 kartų per dieną<br>○ 11 kartų per dieną arba dažniau
Kava be kofeino (150ml)	○ niekada<br>○ 1 kartą per mėnesį arba rečiau<br>○ 2-3 kartus per mėnesį<br>○ 1-3 kartus per savaitę<br>○ 4-6 kartus per savaitę<br>○ 1-2 kartus per dieną	○ 3-4 kartus per dieną<br>○ 5-6 kartus per dieną<br>○ 7-8 kartus per dieną<br>○ 9-10 kartų per dieną<br>○ 11 kartų per dieną arba dažniau
Juoda arba žalia arbata (150ml)	○ niekada<br>○ 1 kartą per mėnesį arba rečiau<br>○ 2-3 kartus per mėnesį<br>○ 1-3 kartus per savaitę<br>○ 4-6 kartus per savaitę<br>○ 1-2 kartus per dieną	○ 3-4 kartus per dieną<br>○ 5-6 kartus per dieną<br>○ 7-8 kartus per dieną<br>○ 9-10 kartų per dieną<br>○ 11 kartų per dieną arba dažniau

Vaisinė arba žolelių arbata (150ml)	○ niekada<br>○ 1 kartą per mėnesį arba rečiau<br>○ 2-3 kartus per mėnesį<br>○ 1-3 kartus per savaitę<br>○ 4-6 kartus per savaitę<br>○ 1-2 kartus per dieną	○ 3-4 kartus per dieną<br>○ 5-6 kartus per dieną<br>○ 7-8 kartus per dieną<br>○ 9-10 kartų per dieną<br>○ 11 kartų per dieną arba dažniau

Kaip Jūs paprastai geriate kavą arba arbatą?
(Galima pateikti kelis atsakymo variantus)

Kava	○ negeriu<br>○ su kondensuotu pienu	○ juoda<br>○ su cukrumi	○ su pienu<br>○ su saldikliu
Juoda, žalia arbata	○ negeriu<br>○ su medumi	○ be priedų<br>○ su cukrumi	○ su pienu<br>○ su saldikliu
Vaisinė, žolelių arbata	○ negeriu<br>○ su cukrumi	○ be priedų<br>○ su saldikliu	○ su medumi

Alkoholiniai gėrimai

Kaip dažnai Jūs pastaruosius 12 mėnesių vartojote žemiau išvardintus alkoholinius gėrimus? Prašome nurodyti ir įprastinį alkoholinio gėrimo kiekį (porciją)! Atkreipkite dėmesį į alkoholinių gėrimų vartojimą nedarbo bei švenčių dienomis.

Alus	○ niekada<br>○ 1 kartą per mėnesį arba rečiau<br>○ 2-3 kartus per mėnesį<br>○ 1 kartą per savaitę<br><br>Porcija: 0,5 l butelis □□□	○ 2-3 kartus per savaitę<br>○ 4-6 kartus per savaitę<br>○ 1 kartą per dieną<br>○ 2 kartus per dieną arba dažniau
Nealkoholinis alus	○ niekada<br>○ 1 kartą per mėnesį arba rečiau<br>○ 2-3 kartus per mėnesį<br>○ 1 kartą per savaitę<br><br>Porcija: 0,5 l butelis □□□	○ 2-3 kartus per savaitę<br>○ 4-6 kartus per savaitę<br>○ 1 kartą per dieną<br>○ 2 kartus per dieną arba dažniau
Baltas vynas	○ niekada<br>○ 1 kartą per mėnesį arba rečiau<br>○ 2-3 kartus per mėnesį<br>○ 1 kartą per savaitę<br><br>Porcija: 0,25 l taurė □□□	○ 2-3 kartus per savaitę<br>○ 4-6 kartus per savaitę<br>○ 1 kartą per dieną<br>○ 2 kartus per dieną arba dažniau
Raudonas vynas	○ niekada<br>○ 1 kartą per mėnesį arba rečiau<br>○ 2-3 kartus per mėnesį<br>○ 1 kartą per savaitę<br><br>Porcija: 0,25 l taurė □□□	○ 2-3 kartus per savaitę<br>○ 4-6 kartus per savaitę<br>○ 1 kartą per dieną<br>○ 2 kartus per dieną arba dažniau

Vaisių vynas	○ niekada<br>○ 1 kartą per mėnesį arba rečiau<br>○ 2-3 kartus per mėnesį<br>○ 1 kartą per savaitę	○ 2-3 kartus per savaitę<br>○ 4-6 kartus per savaitę<br>○ 1 kartą per dieną<br>○ 2 kartus per dieną arba dažniau
	Porcija: 0,25 l taurė □□□	
Putojantis vynas	○ niekada<br>○ 1 kartą per mėnesį arba rečiau<br>○ 2-3 kartus per mėnesį<br>○ 1 kartą per savaitę	○ 2-3 kartus per savaitę<br>○ 4-6 kartus per savaitę<br>○ 1 kartą per dieną<br>○ 2 kartus per dieną arba dažniau
	Porcija: 0,1 l taurė □□□	
Likeris, aperityvas (pvz. cheresas, portveinas)	○ niekada<br>○ 1 kartą per mėnesį arba rečiau<br>○ 2-3 kartus per mėnesį<br>○ 1 kartą per savaitę	○ 2-3 kartus per savaitę<br>○ 4-6 kartus per savaitę<br>○ 1 kartą per dieną<br>○ 2 kartus per dieną arba dažniau
	Porcija: 80 ml taurė □□□	
Desertinis vynas	○ niekada<br>○ 1 kartą per mėnesį arba rečiau<br>○ 2-3 kartus per mėnesį<br>○ 1 kartą per savaitę	○ 2-3 kartus per savaitę<br>○ 4-6 kartus per savaitę<br>○ 1 kartą per dieną<br>○ 2 kartus per dieną arba dažniau
	Porcija: 0,1 l taurė □□□	
Spiritiniai gėrimai (pvz. brendis, degtinė)	○ niekada<br>○ 1 kartą per mėnesį arba rečiau<br>○ 2-3 kartus per mėnesį<br>○ 1 kartą per savaitę	○ 2-3 kartus per savaitę<br>○ 4-6 kartus per savaitę<br>○ 1 kartą per dieną<br>○ 2 kartus per dieną arba dažniau
	Porcija: 20 ml taurė □□□	
Alkoholiniai kokteiliai (pvz. radleris, pina-colada ir t.t.)	○ niekada<br>○ 1 kartą per mėnesį arba rečiau<br>○ 2-3 kartus per mėnesį<br>○ 1 kartą per savaitę	○ 2-3 kartus per savaitę<br>○ 4-6 kartus per savaitę<br>○ 1 kartą per dieną<br>○ 2 kartus per dieną arba dažniau
	Porcija: 0,2 l taurė □□□	

Riebalai

Kokio riebumo buvo Jūsų vartoti (žemiau nurodyti) produktai?

Pienas	○ nežinau/įvairus<br>○ 0,3% riebumo	○ 1,5% riebumo<br>○ 3,5% riebumo
Jogurtas	○ nežinau/įvairus<br>○ 0,3% riebumo	○ 1,5% riebumo<br>○ 3,5% riebumo

Minkštas pelėsinis sūris	○ nežinau/įvairus	○ liesas	○ įprastinio riebumo
Olandiškas sūris	○ nežinau/įvairus	○ liesas	○ įprastinio riebumo
Mėsa	○ nežinau/įvairi	○ liesa	○ įprastinio riebumo
Dešra	○ nežinau/įvairi	○ liesa	○ įprastinio riebumo

Riebalai ir aliejai

Kaip dažnai Jūsų valgytų mėsos ar žuvies patiekalų gaminimui buvo naudojami žemiau išvardinti riebalai arba aliejai?

Sviestas	○ niekada	○ retai	○ dažnai	○ visada	○ nežinau /įvairiai
Margarinas	○ niekada	○ retai	○ dažnai	○ visada	○ nežinau /įvairiai
Augaliniai riebalai (pvz. kokoso riebalai)	○ niekada	○ retai	○ dažnai	○ visada	○ nežinau /įvairiai
Gyvuliniai riebalai (pvz. lydyti taukai, lašiniai)	○ niekada	○ retai	○ dažnai	○ visada	○ nežinau /įvairiai
Alyvuogių aliejus	○ niekada	○ retai	○ dažnai	○ visada	○ nežinau /įvairiai
Linų sėmenų aliejus	○ niekada	○ retai	○ dažnai	○ visada	○ nežinau /įvairiai
Saulėgrąžų, usnių, daigų arba kitoks aliejus	○ niekada	○ retai	○ dažnai	○ visada	○ nežinau /įvairiai

Kaip dažnai Jūsų valgytų daržovių patiekalų gaminimui buvo naudojami žemiau išvardinti riebalai/aliejai arba kaip dažnai daržovės buvo jais gardinamos?

Sviestas	○ niekada	○ retai	○ dažnai	○ visada	○ nežinau /įvairiai
Margarinas	○ niekada	○ retai	○ dažnai	○ visada	○ nežinau /įvairiai
Augaliniai riebalai (pvz. kokoso riebalai)	○ niekada	○ retai	○ dažnai	○ visada	○ nežinau /įvairiai

Gyvuliniai riebalai (pvz. lydyti taukai, lašiniai)	○ niekada	○ retai	○ dažnai	○ visada	○ nežinau /įvairiai
Alyvuogių aliejus	○ niekada	○ retai	○ dažnai	○ visada	○ nežinau /įvairiai
Linų sėmenų aliejus	○ niekada	○ retai	○ dažnai	○ visada	○ nežinau /įvairiai
Saulėgrąžų, usnių, daigų arba kitoks aliejus	○ niekada	○ retai	○ dažnai	○ visada	○ nežinau /įvairiai

Padažai

Kaip dažnai Jūsų valgyti žemiau išvardinti maisto produktai buvo gardinami padažais?

Mėsa arba žuvis	○ niekada	○ retai	○ dažnai	○ visada	○ nežinau /įvairiai
Daržovės	○ niekada	○ retai	○ dažnai	○ visada	○ nežinau /įvairiai
Salotos arba žalios (nevirtos) daržovės	○ niekada	○ retai	○ dažnai	○ visada	○ nežinau /įvairiai

Salotų pagardai

Kaip dažnai gardinate salotas šiais priedais?

Alyvuogių aliejus	○ niekada	○ retai	○ dažnai	○ visada	○ nežinau /įvairiai
Linų sėmenų aliejus	○ niekada	○ retai	○ dažnai	○ visada	○ nežinau /įvairiai
Saulėgrąžų, usnių, daigų arba kitoks aliejus	○ niekada	○ retai	○ dažnai	○ visada	○ nežinau /įvairiai
Majonezu	○ niekada	○ retai	○ dažnai	○ visada	○ nežinau /įvairiai
Grietine	○ niekada	○ retai	○ dažnai	○ visada	○ nežinau /įvairiai
Jogurtu	○ niekada	○ retai	○ dažnai	○ visada	○ nežinau /įvairiai
Žaliomis žolelėmis	○ niekada	○ retai	○ dažnai	○ visada	○ nežinau /įvairiai

Porcijos

Prašome pagalvoti apie įprastinę savaitę šaltuoju pusmečiu (nuo spalio iki kovo mėn.) arba šiltuoju pusmečiu (nuo balandžio iki rugsėjo mėn.). Kiek porcijų žemiau nurodytų Jūs suvalgydavote per savaitę? (Mažiau nei viena porcijas per savaitę = 0). Pavyzdys: Jūs suvalgydavote 3 porcijas vaisių per dieną. Tai reiškia, jog per savaitę Jūs suvalgydavote 3 x 7 = 21 porcijas vaisių.

Virtų daržovių	pavasarį/vasarą □□ porcijas per savaitę<br>rudenį/žiemą □□ porcijas per savaitę
Salotų ir žalių (nevirtų) daržovių	pavasarį/vasarą □□ porcijas per savaitę<br>rudenį/žiemą □□ porcijas per savaitę
Vaisių	pavasarį/vasarą □□ porcijas per savaitę<br>rudenį/žiemą □□ porcijas per savaitę

Prašome pagalvoti apie įprastinę pastarųjų 12 mėnesių savaitę. Kiek porcijų žemiau išvardintų produktų Jūs vidutiniškai suvalgydavote per savaitę? (Mažiau nei viena porcija per savaitę = 0). Pavyzdys: Jūs kas 2-3 dienas suvalgydavote po 1 porciją sūrio. Tai reiškia, jog per savaitę Jūs suvalgydavote 7/2,5 = 3 porcijos sūrio.

Sūrio	□□ porcijas per savaitę
Dešros	□□ porcijas per savaitę
Mėsos (įskaitant kotletus, mėsainius, keptas dešreles)	□□ porcijas per savaitę
Saldumynų (šokolado, saldainių)	□□ porcijas per savaitę
Kepinių	□□ porcijas per savaitę
Šilto maisto	□□ porcijas per savaitę

Fizinis aktyvumas

Pagalvokite apie savo įprastinę savaitę ir nurodykite, kiek valandų per savaitę Jūs per pastaruosius 12 mėnesių vykdėte žemiau išvardintas veiklas šaltuoju pusmečiu (nuo spalio iki kovo mėn.) arba šiltuoju pusmečiu (nuo balandžio iki rugsėjo mėn.). Kiek valandų per savaitę Jūs vidutiniškai skyrėte šioms veikloms?

Ėjimui pėsčiomis, pasivaikščiojimui, ėjimui į darbą, apsipirkti	pavasarį/vasarą □□ porcijas per savaitę<br>rudenį/žiemą □□ porcijas per savaitę
Važiavimui dviračiu	pavasarį/vasarą □□ porcijas per savaitę<br>rudenį/žiemą □□ porcijas per savaitę
Sportui (išskyrus važiavimui dviračiu)	pavasarį/vasarą □□ porcijas per savaitę<br>rudenį/žiemą □□ porcijas per savaitę
Darbui sode	pavasarį/vasarą □□ porcijas per savaitę<br>rudenį/žiemą □□ porcijas per savaitę

Kiek valandų per savaitę Jūs vidutiniškai skyrėte šioms veikloms?

Darbui, skirtam namo ar buto priežiūrai (remontui, taisymui)	□□ valandų per savaitę
Namų ruošos darbams (virimui, skalbimui, valymui)	□□ valandų per savaitę

Ar per pastaruosius 12 mėnesių vykdėte šias veiklas? Jeigu taip, kiek vidutiniškai per dieną?

Lipote laiptais į viršų	□□ aukštų per dieną
Žiūrėjote televiziją	□□ valandų per dieną
Miegojote	dienos metu □□ valandų<br>nakties metu □□ valandų

Pastabos

Jeigu norite pateikti papildomų duomenų apie savo mitybą, galite įrašyti juos į greta esantį teksto laukelį.	□□□□□□□□□□□□□□□□□□□□□□□□<br>□□□□□□□□□□□□□□□□□□□□□□□□<br>□□□□□□□□□□□□□□□□□□□□□□□□<br>□□□□□□□□□□□□□□□□□□□□□□□□<br>□□□□□□□□□□□□□□□□□□□□□□□□

Dėkojame už Jūsų bendradarbiavimą!
