# Supplementary figures and images for: Gut microbial similarity in twins is driven by shared environment and aging
Source: eBioMedicine. 2022 Apr 29;79:104011. doi: 10.1016/j.ebiom.2022.104011 (PMC9062754; doi:10.1016/j.ebiom.2022.104011)

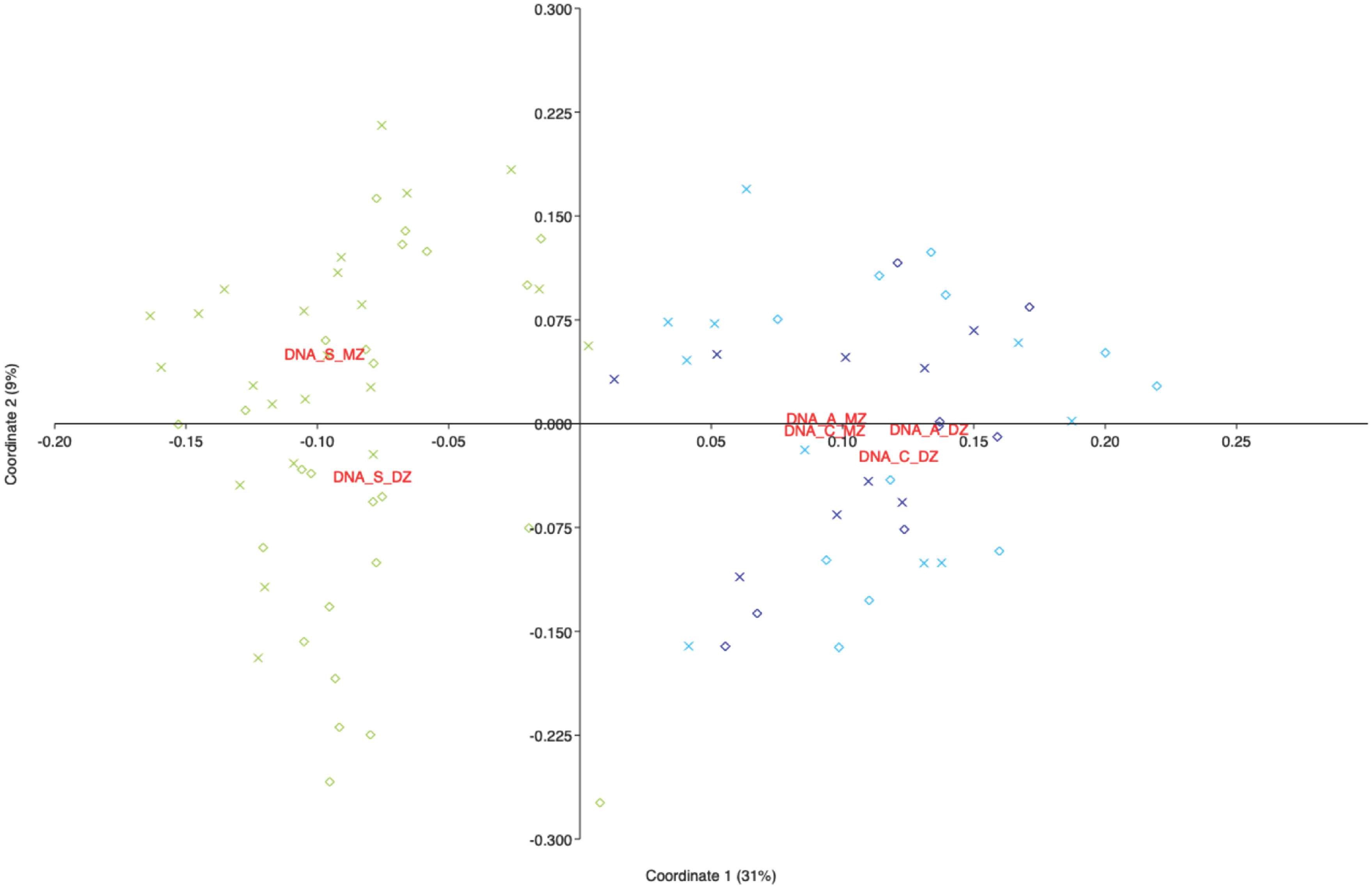

Supplement: Supplementary file 5 [file mmc5.jpg]

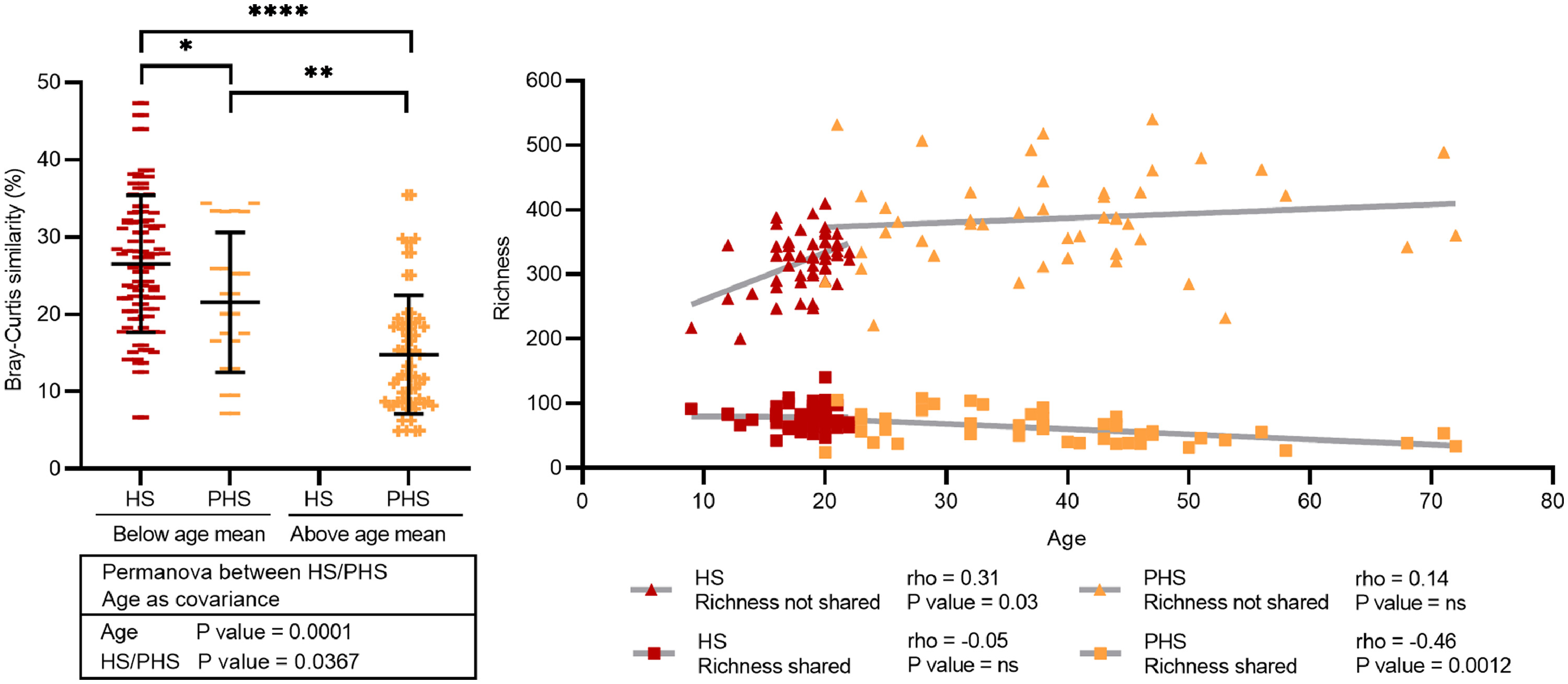

Supplement: Supplementary file 9 [file mmc9.jpg]

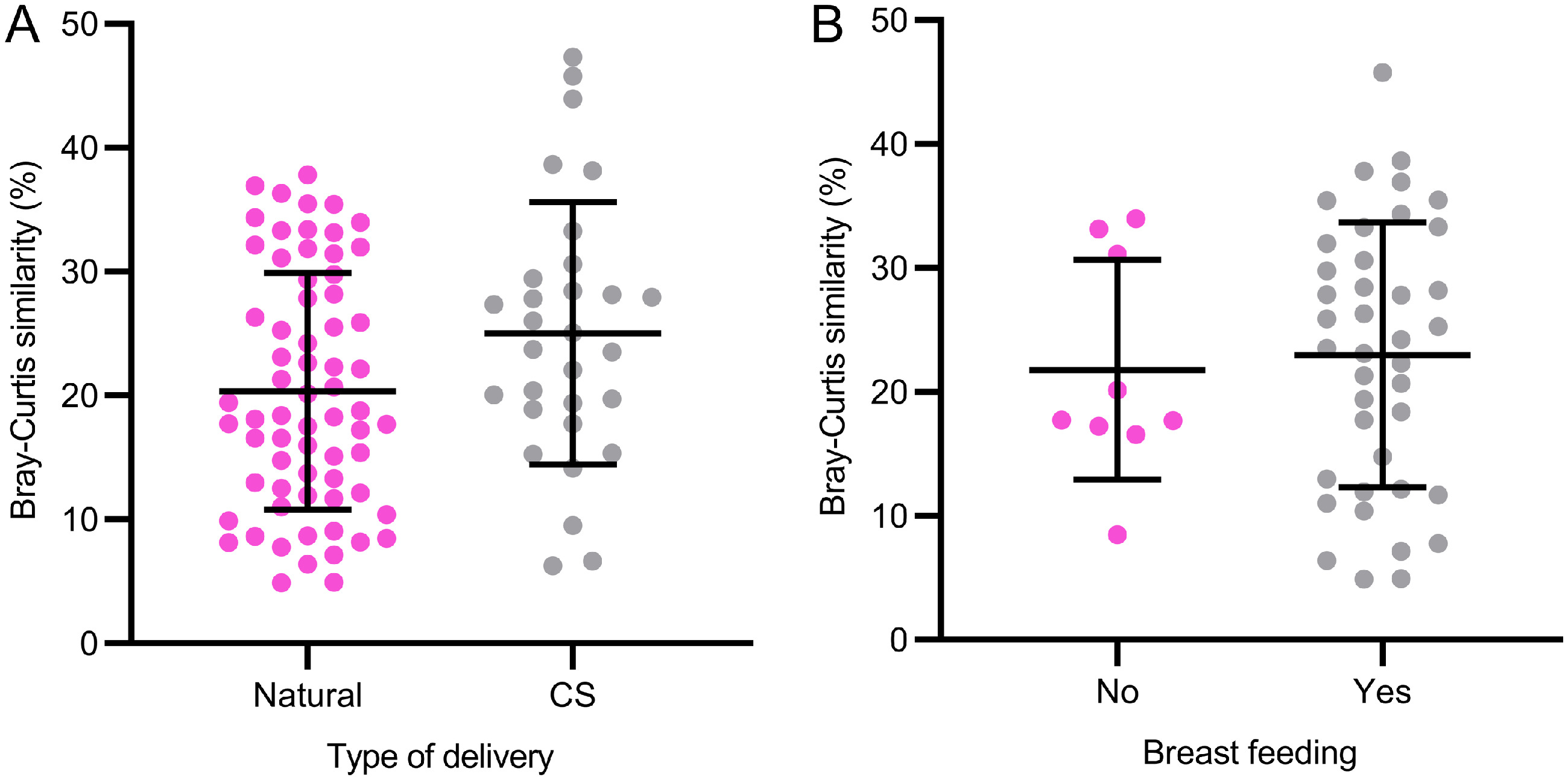

Supplement: Supplementary file 10 [file mmc10.jpg]
