# Supplementary material for: Gut microbial similarity in twins is driven by shared environment and aging
Source: eBioMedicine. 2022 Apr 29;79:104011. doi: 10.1016/j.ebiom.2022.104011 (PMC9062754; doi:10.1016/j.ebiom.2022.104011)
Supplement: Supplementary file 6 [file mmc6.pdf]

Supplementary Table 6. Genetic and environmental contribution to the most abundant bacteria genera found in the GUT of the cohort.

Trait	TWINUK	A (95% CI)	C (95% CI)	E (95% CI)	P_{FDR} (ACE vs. CE)	P_{FDR} (ACE vs. AE)	P_{FDR} (ACE vs. E)
**Bacteroides**	0.03	0.468 (0-0.64)	0 (0-0.43)	0.532 (0.36-0.76)	0.383	1.00	**$3.10 \cdot 10^{-3}$**
**Blautia**	0.3	0.445 (0-0.65)	0.031 (0-0.48)	0.524 (0.35-0.76)	0.394	1.00	**$3.10 \cdot 10^{-3}$**
**Faecalibacterium**	0.2	0.346 (0-0.56)	0 (0-0.36)	0.654 (0.44-0.91)	0.394	1.00	0.057
**Parabacteroides**	0.1	0 (0-0.39)	0.139 (0-0.33)	0.861 (0.61-1)	1.00	1.00	0.478
**Collinsella**	0.1	0.085 (0-0.55)	0.282 (0-0.51)	0.633 (0.44-0.83)	0.929	1.00	**$5.48 \cdot 10^{-3}$**
**Alistipes**	0	0.354 (0-0.56)	0 (0-0.34)	0.646 (0.44-0.89)	0.393	1.00	**0.043**
**Ruminococcus**	0.03	0.418 (0-0.62)	0 (0-0.33)	0.582 (0.38-0.84)	0.383	1.00	**0.019**
**Bifidobacterium**	0.31	0.205 (0-0.43)	0 (0-0.24)	0.796 (0.57-1)	0.394	1.00	0.367
**Prevotella**	0.1	0.407 (0.16-0.61)	0 (0-0.34)	0.593 (0.39-0.84)	0.383	1.00	**0.019**
**Holdemanella**	-	0 (0-0.50)	0.381 (0.20-0.54)	0.619 (0.46-0.80)	1.00	1.00	**$3.10 \cdot 10^{-3}$**
**Clostridium IV**	0.24	0.063 (0-0.32)	0 (0-0.20)	0.938 (0.67-1)	0.841	1.00	0.904
**Streptococcus**	0	0.163 (0-0.40)	0 (0-0.30)	0.837 (0.60-1)	0.841	1.00	0.525
**Dialister**	0.1	0.15 (0-0.39)	0 (0-0.28)	0.85 (0.61-1)	0.432	1.00	0.567
**Parasutterella**	-	0.299 (0.04-0.53)	0 (0-0.27)	0.7 (0.47-0.96)	0.375	1.00	0.123
**Catenibacterium**	-	0.259 (0-0.57)	0.113 (0-0.46)	0.628 (0.43-0.87)	0.686	1.00	**0.017**

Reported A, C and E estimates are from the ACE model (A = additive genetic influence; C = shared environmental influence; E = nonshared environmental influence), P-value for A (likelihood ratio test comparing the ACE model to the CE model), P-value for C (likelihood ratio test comparing the ACE model to the AE model), and P-value for E (likelihood ratio test comparing the ACE model to the E model). $P_{FDR} < 0.05$ are presented in **bold**. TWINUK – data from the Goodrich et al. 2016.
