# Supplementary material for: Gut microbial similarity in twins is driven by shared environment and aging
Source: eBioMedicine. 2022 Apr 29;79:104011. doi: 10.1016/j.ebiom.2022.104011 (PMC9062754; doi:10.1016/j.ebiom.2022.104011)
Supplement: Supplementary file 7 [file mmc7.pdf]

Supplementary file 7: Results of the regression analysis for four known confounding factors on the box-cox transformed relative abundances of the 18 predominant bacteria taxa. Abbreviations: Std. Error - standard error of the regression, Pr(>|t|) - the p-value associated with the value in the t value column. P-values <0.05 are marked in bold.

		gender	age	living together	total sequencing read number
Bacteroides	Estimate	0,0320	2,73E-04	0,1296	-6,95E-07
	Std. Error	0,0164	2,61E-03	0,0601	2,76E-06
	t value	1,9560	0,1050	2,1550	-0,2520
	Pr(>|t|)	0,0519	0,9170	**0,0394**	0,8010
Blautia	Estimate	-0,2616	-7,50E-03	2,33E-03	2,14E-05
	Std. Error	0,1299	6,93E-03	0,1989	7,34E-06
	t value	-2,0140	-1,0820	0,0120	2,9180
	Pr(>|t|)	**0,0454**	0,2807	0,9907	**3,94E-03**
Faecalibacterium	Estimate	0,0116	-0,0019	0,0136	6,93E-07
	Std. Error	6,07E-03	1,08E-03	0,0310	3,56E-07
	t value	1,9170	-1,7280	0,4380	1,9480
	Pr(>|t|)	0,0567	0,0856	0,6616	0,0529
Parabacteroides	Estimate	-0,0385	2,55E-03	0,0373	4,69E-07
	Std. Error	0,0322	1,03E-03	0,0186	1,82E-06
	t value	-1,1970	2,4840	2,0110	0,2580
	Pr(>|t|)	0,2330	**0,0138**	**0,0457**	0,7970
Ruminococcus	Estimate	-0,0715	7,00E-03	0,0111	-4,34E-07
	Std. Error	0,0411	3,91E-03	0,0630	2,32E-06
	t value	-1,7380	1,7900	0,1760	-0,1870
	Pr(>|t|)	0,0838	0,0750	0,8605	0,8522
Alistipes	Estimate	-0,0694	1,40E-03	0,1376	4,12E-06
	Std. Error	0,0429	7,30E-04	0,0658	2,43E-06
	t value	-1,6170	1,9140	2,0920	1,6990
	Pr(>|t|)	0,1075	0,0571	**0,0377**	0,0910
Collinsella	Estimate	-0,0894	-9,03E-03	-0,1830	6,20E-06
	Std. Error	0,0744	3,97E-03	0,1140	4,21E-06
	t value	-1,2020	-2,2740	-1,6060	1,4750
	Pr(>|t|)	0,2310	**0,0240**	0,1100	0,1420
Bifidobacterium	Estimate	5,23E-03	-9,70E-03	0,1126	-2,73E-06
	Std. Error	0,0822	4,39E-03	0,0528	1,44E-06
	t value	0,0640	-2,2100	2,1330	-1,8970
	Pr(>|t|)	0,9493	**0,0283**	**0,0342**	0,0593
Prevotella	Estimate	0,3739	-1,96E-03	-0,0111	1,84E-05
	Std. Error	0,2011	0,0107	0,3079	1,14E-05
	t value	1,8600	-0,1830	-0,0360	1,6230
	Pr(>|t|)	0,0645	0,8553	0,9714	0,1063
Streptococcus	Estimate	-2,1660	-0,1936	-5,0620	1,71E-04
	Std. Error	2,0150	0,1075	3,0850	6,83E-05

	t value	-1,0750	-1,8000	-1,6410	2,5000
	Pr(>|t|)	0,2836	0,0733	0,1025	**0,0133**
Holdemanella	Estimate	-0,3629	0,0164	0,0347	-1,16E-05
	Std. Error	0,1655	0,0162	0,4657	5,89E-06
	t value	-2,1930	1,0070	0,0750	-1,9760
	Pr(>|t|)	**0,0295**	0,3150	0,9410	**0,0496**
Clostridium.IV	Estimate	-0,5790	0,0597	0,8512	3,30E-06
	Std. Error	0,2546	0,0243	0,6958	2,57E-05
	t value	-2,2740	2,4600	1,2230	0,1280
	Pr(>|t|)	**0,0241**	**0,0148**	0,2227	0,8980
Catenibacterium	Estimate	0,5403	0,0165	-0,2940	2,13E-05
	Std. Error	0,2683	0,0373	1,0690	3,95E-05
	t value	2,0140	0,4420	-0,2750	0,5400
	Pr(>|t|)	**0,0454**	0,6590	0,7840	0,5900
Dialister	Estimate	0,1338	-3,69E-03	-0,1296	6,81E-05
	Std. Error	1,1100	0,0593	1,7010	3,43E-05
	t value	0,1210	-0,0620	-0,0760	1,9840
	Pr(>|t|)	0,9040	0,9500	0,9390	**0,0487**
Parasutterella	Estimate	0,2925	0,2935	11,0700	2,21E-04
	Std. Error	7,6810	0,4099	5,1681	4,34E-04
	t value	0,0380	0,7160	2,1420	0,5090
	Pr(>|t|)	0,9700	0,4750	**0,0334**	0,6110
Succinivibrio	Estimate	15950,0	1974,0	1913,0	-2,1380
	Std. Error	30700,0	895,2	47010,0	1,0787
	t value	0,5200	2,2050	0,0410	-1,9820
	Pr(>|t|)	0,6040	**0,0286**	0,9680	**0,0488**
Butyrivibrio	Estimate	-2201,0	-4,5360	-4829,0	-0,2263
	Std. Error	909,1	82,7	2372,0	0,0875
	t value	-2,4210	-0,0550	-2,0360	-2,5850
	Pr(>|t|)	**0,0164**	0,9563	**0,0431**	**0,0105**
Mitsuokella	Estimate	-235,7	86,8	611,0	0,0689
	Std. Error	3211,0	46,2	4918,0	0,1815
	t value	-0,0730	1,8770	0,1240	0,3800
	Pr(>|t|)	0,9420	0,0620	0,9010	0,7050
