# Supplementary material for: Gut microbial similarity in twins is driven by shared environment and aging
Source: eBioMedicine. 2022 Apr 29;79:104011. doi: 10.1016/j.ebiom.2022.104011 (PMC9062754; doi:10.1016/j.ebiom.2022.104011)
Supplement: Supplementary file 8 [file mmc8.pdf]

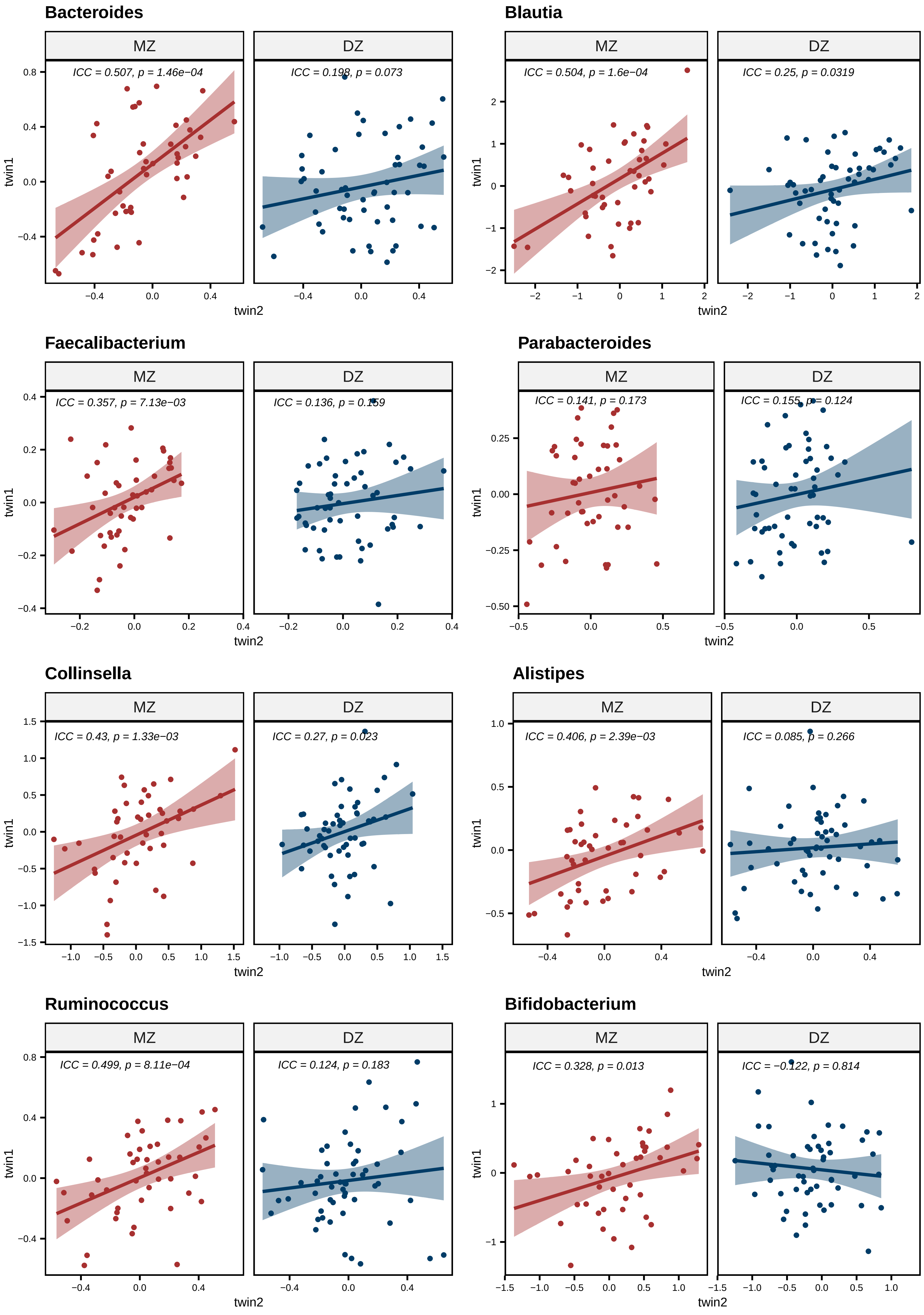
Bacteroides
MZ
DZ
ICC = 0.507, p = 1.46e−04
ICC = 0.198, p = 0.073
Blautia
MZ
DZ
ICC = 0.504, p = 1.6e−04
ICC = 0.25, p = 0.0319
Faecalibacterium
MZ
DZ
ICC = 0.357, p = 7.13e−03
ICC = 0.136, p = 0.159
Parabacteroides
MZ
DZ
ICC = 0.141, p = 0.173
ICC = 0.155, p = 0.124
Collinsella
MZ
DZ
ICC = 0.43, p = 1.33e−03
ICC = 0.27, p = 0.023
Alistipes
MZ
DZ
ICC = 0.406, p = 2.39e−03
ICC = 0.085, p = 0.266
Ruminococcus
MZ
DZ
ICC = 0.499, p = 8.11e−04
ICC = 0.124, p = 0.183
Bifidobacterium
MZ
DZ
ICC = 0.328, p = 0.013
ICC = −0.122, p = 0.814
twin1
twin2

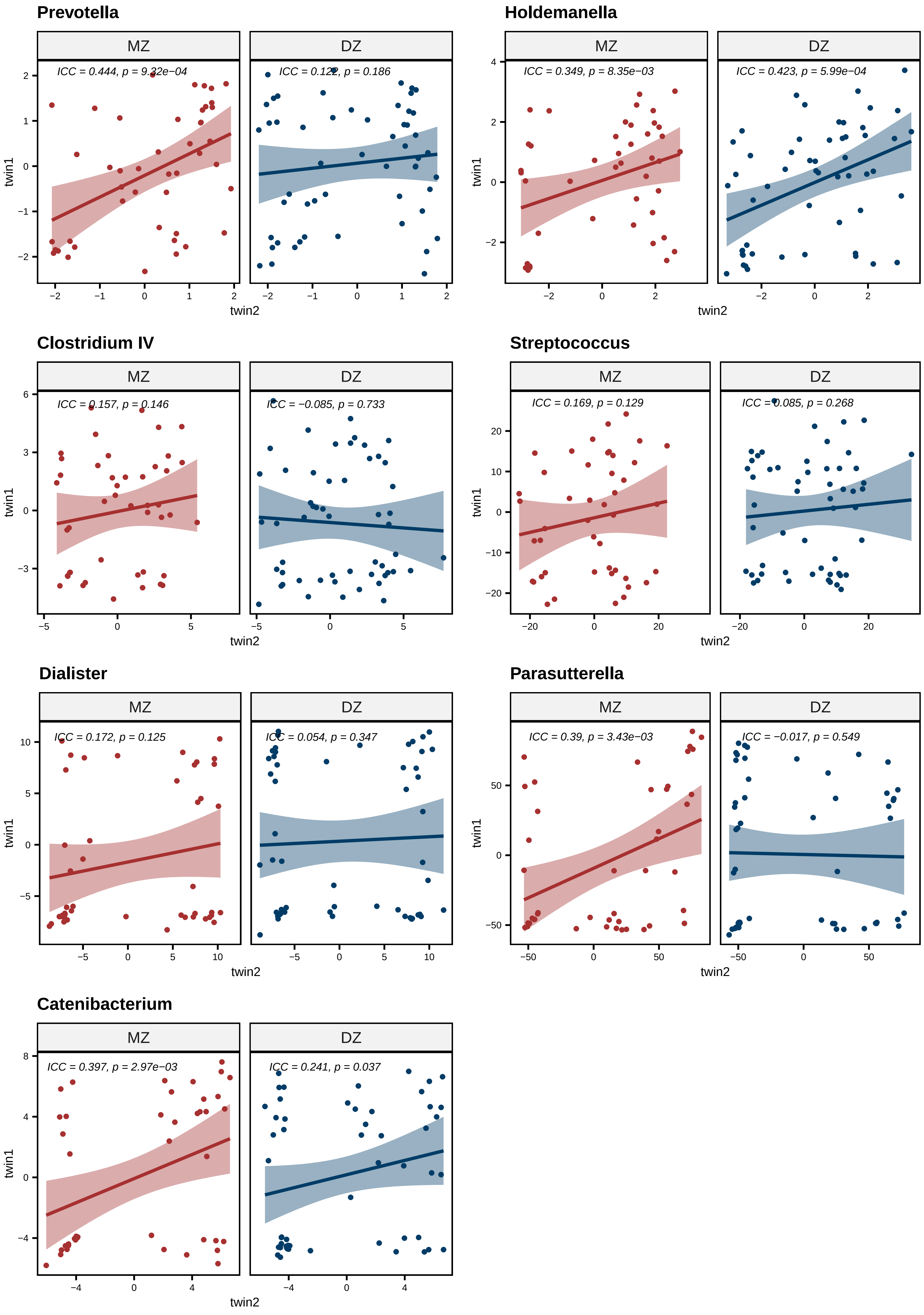

Prevotella
MZ
DZ
ICC = 0.444, p = 9.32e−04
ICC = 0.122, p = 0.186
Holdemanella
MZ
DZ
ICC = 0.349, p = 8.35e−03
ICC = 0.423, p = 5.99e−04
Clostridium IV
MZ
DZ
ICC = 0.157, p = 0.146
ICC = −0.085, p = 0.733
Streptococcus
MZ
DZ
ICC = 0.169, p = 0.129
ICC = 0.085, p = 0.268
Dialister
MZ
DZ
ICC = 0.172, p = 0.125
ICC = 0.054, p = 0.347
Parasutterella
MZ
DZ
ICC = 0.39, p = 3.43e−03
ICC = −0.017, p = 0.549
Catenibacterium
MZ
DZ
ICC = 0.397, p = 2.97e−03
ICC = 0.241, p = 0.037
twin1
twin2
